# American Medical Biography, Etc.

**Published:** 1860-03

**Authors:** 


### NORTH AMERICAN

### MEDICO-CHIRURGICAL REVIEW.

**MARCH, 1860.**

### Analytical and Critical Reviews.

ART. I.—*American Medical Biography, etc. etc.* To which is prefixed a Succinct History of Medical Science in the United States, from the First Settlement of the Country. By JAMES THACHER, M.D. Boston, 1828.

*Some Account of the Pennsylvania Hospital, from its First Rise to the Beginning of the Fifth Month, called May,* 1754. Continuation of the Account of the Pennsylvania Hospital, from the First of May, 1754, to the Fifth of May, 1761.

*Address to the Medical Graduates of the University of Pennsylvania, delivered March* 26, 1835. By GEORGE B. WOOD, M.D., Professor of Materia Medica and Pharmacy in the University. 1836.

*General Catalogue of the Medical Graduates of the University of Pennsylvania, with an Historical Sketch of the Origin, Progress, and Recent State of the Medical Department.* Third edition. 1845.

*Philadelphia and her Medical Schools.* [Seven Articles in the Philadelphia North American and United States Gazette, September 6th,—October 22d, 1856.]

*An Exposition of the Laws which relate to the Medical Profession in England, etc. etc.* By JOHN DAVIES, M.D. London, 1844.

ABANDONING, for the nonce, our office of critic, that we may take the more grateful one of historian, we propose to deduce, from the materials found in the productions whose titles head this article, a narrative of the origin and present means of medical instruction in Philadelphia. The first medical school in the then British colonies, which in a few years after became the United States of North America, was established in this city. This event, followed after a short period the founding of the Pennsylvania Hospital, also the first institution, in point of date, of the kind. These, compared with analogous undertakings in other places—New York and Boston for example—would, on account of their prior origin, give some advantage in the competitive struggle for subsequent advance and distinction. But the vantage ground uninterruptedly maintained by Philadelphia, and more conspicuous at the present than at any former period, must be explained by the admission of other causes than of greater age. Not a little is owing to the impetus and direction imparted to the first medical school by its founders, those who were at the same time charged with its early guidance. The plan was not thrown off at a single heat: it was the result of much previous thought and reflection. Nor was the construction hurried, like an extempore palace of ice, to gratify imperial whim, but it was gradually completed in proportion as fitting material presented itself. The men had undergone a training to prepare and strengthen themselves for the work, and some of them early volunteered their services and waited for reinforcement from others of their own kind to carry it out. But opportunity, made up of time, place, and certain associated circumstances, must concur with the best devised schemes and most earnest intentions, in order to insure success. In the present case, there was not only the encouraging support of men of good general scholarship, but, also, of well-educated and well-read physicians, in Philadelphia, who, having themselves experienced the great benefits from attendance on medical schools abroad would rejoice to see an institution of the same character at home, even though they might not be inclined to take the initiative in its creation. Among the elements of continued success, it would be wrong to deny, even if we were so inclined, the favoring influence of the City of Philadelphia itself, compounded of geographical position and the character of its inhabitants, on the young men who come here from all parts of the Union, to fill the lecture-rooms of our medical schools. The numerous specific aids and appliances for instruction in both the science and the practice of medicine in this city are not, by any means, peculiar to it; but by a combination of circumstances, to some, perhaps the chief of which we have adverted, they have been more efficient for the purposes intended than in other places in which numbers, talents, and zeal also exist.

A preliminary notice of the earliest associations in England to regulate the practice of medicine will serve to suggest a feeling of gratitude for our escape from many of the evils and abuses under which the people of that country long suffered, an escape attributable to the wisdom and forethought of the founders of the medical schools, first in Philadelphia and New York, and not very long after in Boston.

A few words on the origin of medical colleges in the Old World will not come amiss, as an introduction to our immediate theme. The term college, (*collegium,*) in its primary sense a collection or assembly, conveys no definite idea of the character or pursuits of the persons who compose it, nor of the objects which they propose to themselves in their collective capacity. College, in a particular sense, signifies an assembly for a political, ecclesiastical, or medical purpose, as well as a society of persons engaged in the pursuits of literature, including officers and students. A number of colleges, of separate origin and endowment, and with each its own government, may constitute a university, as in the cases of Oxford and Cambridge, in England. In ancient Rome there were the *collegium pontificum, augurum, septemvirorum,* etc.; and in the modern city there is the College of Cardinals. Germany has a college of electors, and Russia imperial colleges, composed of councils of state, courts or assemblies, intrusted with the administration of the government.*

So far as we can learn, a medical college is a modern institution, which had its origin at the same time with that of universities. Of these, the University of Paris takes precedence in the order of time—the early part of the twelfth century—and in the fame of its professors and the crowds of its students, who, in a spirit of hyperbole, were represented to be more numerous than the citizens. As a school of law the University of Bologna may perhaps lay claim to an earlier origin, as it could afterwards boast of thousands of students in regular attendance in its halls. That of Montpelier acquired early celebrity by its medical school. We ought to speak of the School of Salernum, which was the nursery of medicine for modern Europe, as being of a collegiate character, but we lack the requisite details of its precise organization. We find the more difficulty in investigations of this nature from the double meaning which has been attached to a college, as a corporate medical institution. There are, in fact, two kinds of medical colleges; the one for instructing tyros in the different branches of medicine according to a curriculum duly announced, and for conferring the degree of doctor of medicine and the right of practicing it as a profession on those who have gone through the prescribed course of study, and acquitted themselves satisfactorily in examinations held by the different professors. These latter, in their collective capacity, constitute a "Faculty," which, together with the faculties of theology, law, and the arts, were the first, as they have continued, in most instances, to be the congregate to make up a university. The first organization of faculties took place in Paris, in the year 1259 and 1260,—the teachers of medicine in the latter year following the

* Encyclopedia Americana.

example set them in this respect by the union of the mendicant orders and secular clergy, who formed themselves into a sort of corporation of teachers of theology. The same course was pursued by the teachers of canon law. The first colleges in the universities were those in the buildings of which students, especially poor ones, might live together, under superintendence, without paying for their lodging. In some cases they received also their board gratis, or had still further allowances.

Contemporaneous with the establishment of universities was the origin of the title of doctor. It was first conferred at the celebrated law school of the University of Bologna, some time between the years 1128 and 1137, and when Irnerius (Werner) began to give prelections in law. It was at his persuasion, as chancellor, that the Emperor Lothario II. was induced to introduce the dignity of doctor. From the faculty of law this title passed to that of theology. The faculty in Paris first conferred the degree of doctor of divinity on Peter Lombard, who in 1159 became Bishop of Paris. Some time elapsed before the third faculty, or that of medicine, thought of granting the doctorate; and it was not until 1329, that William Gordenio, of the college at Asti, in Piedmont, was promoted to the dignity of *doctor artium et medicinæ.* The doctorate of philosophy was established last, because the faculty of philosophy was founded the latest. This title, common enough in the universities of Germany, is not known in those of Great Britain or of the United States. As regards the precedence or rank, on the continent of Europe, it runs in the following order—doctor of theology, of law, of medicine, and of philosophy; but in England and the United States, the doctor of laws ranks first, and the doctor of divinity next. The degree of *doctor of music* is conferred at the Universities of Oxford and Cambridge. Haydn received the title from the first of these institutions.*

Having briefly noticed, of the two kinds of colleges of medicine, that one which prepares, by methodical instruction, those who come within its walls, for engaging in the active duties of the profession, we have next to advert to that other kind which consists of an association of physicians with corporate powers, who prescribe the qualifications for admission into their body, and, in some instances, test the fitness of candidates by an examination. Among the privileges of such a college is that of granting liberty of practicing physic to its members alone, or to graduates of certain specified educational colleges of medicine, or of universities of which these institutions form a component part. The most ancient and a still vigorous and powerful institution of this kind is the College of Physicians of London, which was chartered in the

---

* Op. cit.

tenth year of the reign of Henry VIII., (1519.) The necessity of a controlling and governing body for the encouragement and protection of the regular practice of physic had long been keenly felt in England. We subjoin, in a foot-note,* a curious document, in the shape of a petition to

---

* STATUTE.

9 HEN. V.

[*Cited by Sir Wm. Browne, in his Vindication of the College of Physicians.* Quarto, London, 1753.]

*Ex Bundello petitionum de ano*, 9⁰ *H.* 5, *in Parliamento.*

Hey and most mighty prince, noble and worthy lords, spirituelx and temporelx, and worshipfull communes, for so moche as a man hath three things to governe, that is to say soule, body, and worldly goods, the which ought and shulde ben principaly reweled by thre Sciences, that ben divinitie, fisyk, and lawe, the soule by divinitie, the body by fisyk, worldly goods by lawe, and those conynges should be used and practised principally by the most connyng men in the same sciences, and most approved in cases necessaries to encrese of virtue, long life, and gouds of fortune, to the worship of God and comyn profit. But worthi soveraines hit is known to your hey discretion, many uncunning and unaproved in the aforesaid science practiseth, and specialy in fysyk, so that in this realme is every man, be he never so lewed, taking upon him practice y suffered to use it to grete harm and slaughtre of many men, where if no men practiced therein but al only connyng men and approved sufficiently y learned in art, filosofye, and fysyk, as it is kept in other londes and roilmes, then should many man that dyeth for defaute of helpe lyve, and no man perish by unconning. Wherefore pleseth to your exellents wysdomes that ought, after your soule have no entendance to youre body for the causes abovesaid, to ordaine and make in statute perpetually to be strictly y used and kept. That no man of no manner, estate, degre, or condition, practise in fysyk fro this time forward bot he have long time y used the scoles of fysyk, having letters testimonialx sufficianty of on of those degrees in the universite in which he took his degree in, under payne of long impresonement and paying XL lb. to the king, and that no woman use the practice of fysyk under the same payne, and that the sherrifs of every shire make enquisiteon in their tournes if there be any that forfaiteth agens this statuit under a payne reasonable, and thenne that they put this statute in execution without any favoure under the same payne; also lest that they the which ben able to practise in fisyk ben excluded fro practise, the which be not graduated. Pleseth to your hey prudency to send writtes to all the sherrifs of England that, every practysour in fisyk not graduated in the same science, that wole practise forth be wythin on of the universities of this lond by a certain day, that they that ben able move aftre true and straight examination be received to their degree, and that they that be not able to cese fro the practise into the time they ben able and approved, or for to never more entermete thereof, and that herto also be y set a peyne convenient.

*Dorso,*

Responsio hujus petitionis patet in rotulo parliamento dat 2, die maij. anno regni regis Henr. 5ti., post conquestum nono.

Rot. Parl. 9, H. 5, p. 1, No. 11.

King Henry V. and to the lords spiritual and temporal, and the commons, in parliament assembled, (1321,) setting forth the evils complained of, and pointing out the remedy. Women figure early among the irregular practitioners whom it is the object of the petition to restrain. The functions of the three faculties of divinity, law, and physic, are quaintly and curtly told in the petition.* We retain the orthography of the original, as we find it copied into Paris and Fonblanque's Medical Jurisprudence, (vol. iii., appendix.) Following it, in that work, is an ordinance in Norman-French, issued in the same reign, against interlopers in surgery and physic.† It is a literary curiosity, as showing that, at the very time when France was almost entirely subjugated by an English king, Henry V., this prince sent forth an official rescript in the language of his remote ancestor, William the Conqueror, the use of which was continued in the pleadings and decisions of courts of law to the middle of the fourteenth century; thus perpetually reminding the people of England of their being ruled by a dynasty of foreign origin.

Before the act of incorporation of the College of Physicians of London, one of a preliminary character had been passed in the eighth year of the reign of Henry VIII., in which it was enacted that no person within the City of London, nor within seven miles of the same, shall "take upon him to exercise and occupy as a physician or surgeon, except he be first examined, approved and admitted by the bishop of London, or by the dean of St. Paul's, for the time being, calling to him or them four doctors of physic, and for surgery other expert persons in that faculty—under pain of forfeiture by those not so admitted for every month they do occupy as physicians or surgeons." It was further enacted "that no person out of the said city and precinct of seven miles of the same, except he have been (as is aforesaid) approved on the same, take upon him to exercise and occupy, as a physician or surgeon, in any diocese within this realm; but if he be first examined and approved by the bishop of the same diocese, or he being out of the diocese by his vicar-general, either of them calling to them such expert person, in the same faculties, as their discretion shall think convenient." A provision was expressly made, that nothing in this act should be to the prejudice of the Universities of Oxford and Cambridge, or either of them, or of any privileges granted to them.

---

* In the words "conynges" and "connyng," and "lewed,"we find examples of the change of meaning gradually wrought in the English language. Cunning is here used in the sense of knowledge and skill—an honorable acceptation of it admitted centuries later in the authorized version of the Book of Chronicles, xxv. 3. Lewed in the petition means ignorant.

† Lordinance encontre les entremettours de fysyk et de Surgerie.

The petitioners to the king and parliament for statutory regulations of the practice of medicine, complain "forasmuch as the science and cunning of physick and surgery (to the perfect knowledge whereof be requisite both great learning and experience) is daily within this realm exercised by a great multitude of ignorant persons, of whom the great part have no manner of insight in the same, nor in any other kind of learning; some also can no letters on the book, so far forth, that common artificers, as smiths, weavers, and women, boldly and accustomally take upon them great cures, and things of great difficulty, in the which they purely use sorcery and witchcraft, partly apply such medicine to the disease as be very noious, and nothing meet, therefore, to the high displeasure of God, great infamy of the faculty, and grievous hurt, damage, and destruction of many of the king's liege people, most especially of them that cannot discern the uncunning from the cunning." The grievances complained of were no doubt very great, but much cannot be said in favor of the language or the manner in which they were set forth by the petitioners. Little influence of a beneficial kind would seem to have been exerted on the practice of medicine in England by the Universities of Oxford and Cambridge,—whether from the smallness of the number or the practical deficiencies of the graduates whom they sent forth, does not appear. The teachings in these institutions were of necessity confined to scholastic lore, to the neglect of anatomy and clinical medicine, for acquiring a knowledge of which no opportunities could be offered in towns of the limited population of Oxford and Cambridge. Even at the present day, the same wants are felt and nearly the same deficiencies exist in these places. Stronger language can hardly be used than that of two English writers* in the following sentence: "And with respect to the study and practice of physic, it seems probable that, until after the formation of the College of Physicians, it had not even assumed the character and dignity of a regular profession, for we find that the very few learned men in that branch, which the annals of the period can furnish, had acquired their knowledge in the foreign universities."

The college is authorized by its charter to hold meetings and make by-laws, not only for its own good government, superintendence and correction, but also for those of all persons exercising the faculty in the city or within seven miles thereof. The exercise of the practice of physic within the same limits was allowed only to those who had been admitted by the president and college, through letters under their common seal. A penalty of five pounds every month was incurred by any undeserved person who continued to practice. By a subsequent act it was decreed that no

* Paris and Fonblanque, Medical Jurisprudence, vol. i., p. 1.

person from henceforth be suffered to exercise or practice in physic through England until such time as he be examined at London, by the said president and three of the elects,* "except he be a graduate of Oxford or Cambridge, which hath accomplished all things for his form without any grace." The charter further directs that the president and college should, every year, elect four censors, who shall have the superintendence, correction and government of all persons exercising the faculty of medicine in any manner in the city, or within seven miles thereof, with powers to punish for malpractice, and to exercise superintendence and scrutiny of all medicines, and their administration, provided that the punishment should be by fines, amercements, imprisonment, and other reasonable modes. Immunity is given to the president and fellows of the college and their successors, from summons to assizes, juries, inquests, attaints, *et aliis recognitionibus*, by the mayor, sheriffs, or coroners of the city, even by the king's writ. By another act, the members of the college are discharged from keeping watch and ward in the city or its suburbs, and they shall not be chosen to the office of constable or any other office within the same limits. The censors are empowered to enter the houses of apothecaries in the city for the purpose *only* of searching and viewing their wares, drugs, and stuffs, and if any be found defective or corrupted, they may cause them to be burnt, or otherwise destroyed, and a penalty of five pounds, to be recovered by any that will sue for it, is inflicted on apothecaries who obstinately or willfully refuse or deny the four censors to enter into their houses.

There was an important recognition that surgery is included in the science and practice of physic, and hence any member of the college is authorized "to practice and exercise the said science of physic in all and every his members and parts." The candidate must not be less than twenty-six years of age, and must have devoted at least five years to the study of medicine.

A few lines are due to the class of surgeons at this time, and of the difficulties which those of a strictly professional character and attainments had to detach their order from those who had acquired a legal right to thrust themselves into their company. It would seem from the early charters and acts of parliament referred to by Dr. Davies, in his *Exposition*, that there existed in London three classes of surgeons: First, physician-surgeons, or those who practiced medicine and surgery conjointly, and who were, for the most part, graduates of some university. Second, barber-

* The elects consisted of the six persons mentioned in the acts of incorporation, to whom two were subsequently added, to make the number eight. They fill up their own vacancies, and out of their number the president is elected.

surgeons, who did business in surgery in conjunction with barbery. Third, persons who practiced surgery as a distinct art, and who were generally "foreigners," or persons who had been admitted to the freedom of the city. The barbers enjoyed the privilege of incorporation long before the regular surgeons had obtained any advantage of this nature. As early as the first year of the reign of Edward IV. (1461,) "the freemen of the mystery of barbers of the City of London, using the mystery or faculty of surgery," were incorporated into one perpetual community. The functions of this class did not extend beyond "the curing and healing wounds, blows, and other infirmities, as in the letting of blood and drawing of teeth," etc. By an act of the thirty-second year of Henry VIII., the two classes, surgeons and barber-surgeons, were incorporated into one body politic. Up to this time the surgeons had no corporate powers, and were only known as "The Surgeons of London." The title of the new association was "Masters or Governors of the Mystery and Commonalty of Barbers and Surgeons of London." But although the barbers take precedence in the title of the united company, their right to practice surgery is absolutely abolished, if we except the single operation of drawing teeth. This enforced union lasted down to the eighteenth year of the reign of George II. (1745,) when, by act of parliament, the surgeons were released, and obtained a separate charter. Fifty-five years from this time, or in 1800, a charter was granted in the fortieth year of the reign of George III., constituting the surgeons into a college, "by the name of the Royal College of Surgeons in London." In the seventh year of the reign of the present sovereign, Queen Victoria, (1843,) the corporation has had its title changed into that of "The Royal College of Surgeons of England." Similar privileges and powers are enjoyed by the Colleges of Surgeons of Edinburgh and of Dublin. Four years (it was originally six) of study are required of candidates for membership of the College of Surgeons. It gives, of itself, no instruction, nor does it prescribe where this is to be obtained.

The affinity between apothecaries and grocers is not very obvious, although it existed for more than a century in England before the union of the two trades, as they were then regarded, was effected by an act of incorporation in the fourth year of the reign of James I. (1607.) During the period mentioned, in the metropolis, as it is in some rural districts at the present day, and as we have seen it in our own city, the same persons dealt partly in groceries and partly in drugs; but in process of time it became worth their while to give up their whole attention to the pharmaceutical art. In this way a separate class of tradesmen was formed. The union of the stem and offshoots into one body corporate did not, however, last more than nine years, and another charter, giving the apothecaries an independent existence, was granted, partly at the instance of the physicians. This charter was obtained ninety-five years after the establishment of the College of Physicians. The incorporators were associated under the name of the "Master, Wardens, and Society of the Art and Mystery of Apothecaries of the City of London." The conditions on which any person from this time was allowed to keep or furnish an apothecary shop, or to exercise the art or mystery of an apothecary within the limits of the City of London and seven miles beyond it, were, that he should have served *seven years at least* as an apprentice to an apothecary exercising the same art and being free of the same mystery; and after the expiration of this period shall have submitted himself to an examination before a board composed of the master and wardens, assisted by the president of the College of Physicians, or some physician or physicians named by him. By an amended act, passed in 1815, the company is empowered to appoint persons, not less than two in number, who may enter and search any apothecary's shop, "in any part of England or Wales," and destroy any improper drugs or other things belonging to the business of an apothecary they may find therein. The period of apprenticeship was reduced to five years.

Although the title of college is not applied, but only "Society," to the incorporation of apothecaries, yet it so happens that, of the three great incorporated associations of which we have spoken, the last is the only one which lays down a curriculum of medical education for the guidance of its students. We did not deem it necessary to speak of the gradual encroachment by the apothecaries on the regular physicians, in themselves dispensing medicine and prescribing for and visiting the sick. A long contest between the parties was carried on, which ended by judicial decision in favor of the apothecaries. A more animated strife was afterwards engaged in between the latter and the chemists and druggists, now called "pharmaceutists," who, treading on the heels of the apothecaries, at length succeeded in being permitted, if not formally authorized, to dispense the prescriptions of physicians. In addition to the candidate for practicing as an apothecary being required to give testimonials of his having served an apprenticeship of not less than *five years* to an apothecary,* and of his having attained the age of twenty-one years and being of good moral conduct, he must have followed a course of medical study during not less than three winter and two summer sessions; the winter session being understood to extend from the first of October to the middle of April, with a recess of fourteen days at Christmas; and the summer session from the first of May to the thirty-first of July. The branches on which lectures are to be attended are, chemistry, anatomy and

* The original charter must have been modified in this particular.

physiology, materia medica and therapeutics, botany, anatomical demonstrations, dissections, principles and practice of medicine, forensic medicine. Two courses of sixty lectures each, on midwifery and the diseases of women and children, are to be followed in separate sessions; practical midwifery at any time after the conclusion of the first course of midwifery lectures. The student must attend medical practice during the full term of eighteen months; the first twelve months, that is, from the commencement of the second winter session to the commencement of the third winter session, at a recognized hospital, and the remaining six months either at a recognized hospital or a recognized dispensary.

In the northern part of the island (Scotland) the Universities of Glasgow and Edinburgh, the former of which was the much older institution, had their respective medical colleges; but until the middle of the last century they did not exert much influence on either teaching the science or establishing the ethics of medicine. We may soon have to advert to the origin and brilliant career of the school of medicine of Edinburgh. The colleges at Aberdeen until recently had a very questionable, we ought rather to say a bad, name for the reprehensible facility of conferring medical degrees on nearly all applicants who paid for them. An amended state of things, we believe, now exists there.

Were it a part of our design to give the history of medical institutions in Great Britain, we should deem it necessary to state the circumstances under which the London University, afterwards called the "University College," and the "King's College," in the same city, were organized, as well as to make some mention of the existing provincial schools, and of associations formed in connection with the different hospitals in the metropolis. But what we have said thus far, is only intended as an introduction to a notice of the collegiate foundations in medicine, and the means for acquiring a knowledge of its different branches in our own country. Unable as we are, however, at this time to speak of all that has been done, in this line, in different parts of North America, we shall restrict ourselves mainly to a sketch of the origin and progress of the medical institutions of the City of Philadelphia. For what relates to the physicians whose names appear in the early colonial records, we shall be chiefly indebted to the "History of Medicine in the United States," which serves as an introduction to "Thacher's Medical Biography."

With the first colonists and settlers along the Atlantic seaboard, from Massachusetts Bay to Georgia, there came medical men, incited by feelings similar to those which led to the emigration of their countrymen; and we may suppose in many instances by the desire of accompanying their neighbors, friends, and portions of their own immediate families. They brought with them a fair share of the learning and practical knowledge of the time, such as was attainable by the aid of the different medical institutions of which we have just spoken. Some had availed themselves of the opportunities of travel and of study in celebrated schools on the continent of Europe. There were others, again, who appeared in the double capacity of divines and physicians, and made themselves acceptable in both. This union of medicine and theology has ever been common in the earlier and primitive stages of society, in which religious faith and practices precede a knowledge of science and disquisitions on philosophy, and in which they who have the care of souls are called upon to administer to the relief of ills which torment the body. Considerations of a personal nature led some of the non-conforming clergymen, under the fear of ejectment from their livings and the loss of means of support, to prepare themselves for the medical profession as a resource in a day of need. It was not uncommon, apart from any ulterior view of this kind, for students of divinity to read the medical classics and acquire some familiarity with the writings of Hippocrates, Galen, Celsus, etc. When men thus learned came to form pastoral relations with the people of the colonies, they soon discovered that their small congregations were unable to give them a salary equal to their immediate wants; and hence they were fain to add a secular to their ministerial calling. Some taught school, others devoted a part of their time to the practice of medicine.

The sickness with which the first settlers at Plymouth were attacked, soon after their landing, called into early requisition the services of those among them who were endowed with powers of healing and cure. The first physician of whom we have any knowledge, at this period, was Dr. Samuel Fuller, who was one of the company that came over in the first ship, and was, moreover, a deacon in the Rev. John Robinson's church. These zealous and sturdy Puritans, sticklers for the greatest latitude of creed and action in England, and intolerant of the like freedom to those who differed from them in their new homes, lost no time in laying the foundations of a literary institution for the promotion of learning. Only twenty-two years had elapsed from the date of their first landing on a bleak, inhospitable coast, to the holding of a commencement (1642) at the nascent Harvard College, in which Samuel Bellingham and Henry Sallonstall were graduated in the faculty of arts. These gentlemen were afterwards honored with the degree of M.D. at European universities. Leonard Hoar was graduated at Cambridge in 1650, and repaired to England, where he studied medicine. Never, we believe, has a single family furnished as many disciples of Hippocrates as Charles Chauncy, president of Harvard College in 1652. This gentleman received a medical education in England, and had six sons educated at Harvard College, all of whom studied medicine; and, according to the testimony of Dr. Mather, were, in resemblance of their father, eminent physicians. Among those who were both preachers and physicians, mention may be made of Drs. John Fisk, Nathaniel Williams, and Thomas Thacher. The title of surgeon-general, and captain, too, was borne by Matthew Fuller, in the Provincial forces raised in Plymouth Colony, in 1673.

Among the physicians who lived in the early period of English settlement in New York were Drs. Dupey, Dubois, and Nicoll. Cadwallader Colden, Lieutenant-Governor of the Province, was a distinguished physician, and published his opinions on the best method of treating a malignant fever which was very destructive in the city in 1741. Dr. John Bard, whose professional career extended over nearly half a century, wrote an interesting account of a malignant pleurisy which had prevailed in Long Island in 1749, besides some other papers on medical subjects. The first example of dissection for anatomical instruction in the colonies was that of a man who was executed for murder, in 1750. The autopsy was made by Drs. John Bard and Peter Middleton.

Drawing from the same work* which has furnished us with the accounts of the first physicians in Massachusetts and New York, we learn that "in the latter part of the seventeenth and early part of the eighteenth century many learned and enterprising medical men emigrated from Europe, and established themselves in Pennsylvania." Among these we may mention Dr. Thomas Wynn, an eminent Welsh physician, who, after practicing with much reputation in London, came to this country in 1682, with the original settlers. He was accompanied by his brother, who was also a medical man. Contemporary with these gentlemen was Dr. Griffith Owen, who took the lead in the practice of the profession. His death occurred in 1717, the same year in which Dr. Graeme arrived from England, in the company of the governor, Sir William Keith. Dr. Lloyd Zachary, who died in the prime of life, deserves special notice, as one of the founders of the Philadelphia College and of the Pennsylvania Hospital, and a contributor to both. Dr. John Kearsley, known both as physician and surgeon, endowed a hospital for poor widows, and was chiefly instrumental in the erection of Christ Church, Philadelphia. He was the preceptor of Dr. John Redman and Dr. John Bard, of New York. One of the earliest publications on a medical subject, which had yet appeared in Philadelphia, was an essay on the iliac passion, written in 1740, by Dr. Thomas Cadwallader.

Dr. Redman was a graduate of the school of Leyden, at a time when the celebrated Gaubius, the successor and pupil of the still more celebrated

---

* Dr. Thacher's.

Boerhaave, and Albinus were among its professors. He was the first president of the College of Physicians of Philadelphia. Contemporary with Redman were the two Bonds, (Thomas and his brother Phineas,) Cadwallader, and Evans. Dr. Thomas Bond was a native of Maryland; he traveled in Europe, and spent a considerable time in Paris, where he attended the practice of the great hospital, the Hôtel-Dieu. He began his professional career in Philadelphia in 1734; and he may be regarded as the first to acquire distinction in surgery in that city. He has the credit of having originated the idea of the Pennsylvania Hospital, and he was one of the founders of the College of Philadelphia, and the Academy. Dr. Bond, in connection with Franklin, contributed to give form and a name to the American Philosophical Society. Dr. Phineas Bond shares with his older brother Thomas, and with Thomas Hopkinson, the honor of founding the college which was the precursor of the University of Pennsylvania. This gentleman, who had passed a considerable time abroad, and visited Leyden, Paris, Edinburgh, and London, is represented to have been well qualified to judge correctly of any undertaking for the improvement of the medical character of his country. Dr. Thomas Cadwallader was also imbued with the medical lore acquired by study in Europe. His relations to the Hospital, College, and Philosophical Society, especially as one of the physicians of the first-mentioned institution during the long period of thirty years, evince his expanded benevolence and the weight of his professional and literary character in Philadelphia.

A marked event, not more in the history of the benevolent than in that of the medical institutions of Philadelphia, was the foundation of the Pennsyvania Hospital. The initiative in this measure was taken, we have seen, by Dr. Thomas Bond, who was soon and ably seconded in his philanthropic design by Benjamin Franklin. A body of contributors was not long in being formed; and among them we meet with the names of prominent medical men, viz., Drs. Thomas and Phineas Bond, Thomas Cadwallader, William Chanceller, Cadwalader Evans, Richard Farmer, Thomas Graeme, John Kearsley, Sen., and John Kearsley Jr., Samuel Preston Moore, John Redman, William Shippen, Peter Sinmans, and Lloyd Zachary. After a bill had been brought into the House of Representatives of the Province, for founding a hospital, fears were entertained of its miscarriage under the apprehension, on the part of many of the members, "that the expense of paying physicians would eat up the whole of any fund that could be reasonably expected to be raised." This objection was soon removed by an offer from Drs. Lloyd Zachary, Thomas Bond, and Phineas Bond, to attend the hospital gratuitously for three years,—an example which has been followed to the present day; and the bill was passed on the 7th of February, 1751, and received the governor's assent in the May following. A condition for the grant made in the bill, of two thousand pounds toward the founding, building, and furnishing of the hospital, was, that the like sum should be raised by contributions, the yearly interest of which was to be applied to the accommodation of the sick poor in said hospital, free of charge, for diet, attendance, advice, and medicines. This condition was soon complied with, and on the first of July of the same year (1751) a majority of the contributors met at the State House in Philadelphia, and, pursuant to the act, chose twelve managers and a treasurer.* The new managers lost no time in addressing a petition to Thomas Penn and Richard Penn, proprietaries of the Province of Pennsylvania, in which, after reciting the reasons for erecting a hospital, and the action taken on the subject by the General Assembly, they ask that, "as private persons raise a stock to support the Hospital, and the Assembly built the house, so (that all concerned in the Province may share in the honor, merit, and pleasure of promoting so good a work,) the proprietaries will be pleased to favor us with the grant of a piece of ground for the buildings and their necessary accommodations." The spot first indicated by the managers as that which they would prefer was the vacant part of the square between Ninth and Tenth streets from the Delaware, and south of Mulberry (Arch) street; its dimensions being 396 feet east and west, and 360 feet north and south. The proprietaries, responding to the request of the managers of the hospital, made an offer of the larger part of the ground lying north of Race and between Sixth and Seventh Streets, and of which the greater part of Franklin Square is now constituted. This offer was, however, declined by the managers of the hospital, both on account of the onerous conditions on which the grant was made and of the unsuitableness of the spot designated.† Until a building should be erected, the managers

---

* The names of these gentlemen will probably interest our city readers, and we therefore append them. *Managers:* Joshua Crosby, Benjamin Franklin, Thomas Bond, Samuel Hazard, Richard Peters, Israel Pemberton, Jr., Samuel Rhodes, Hugh Roberts, Joseph Morris, John Smith, Evan Morgan, Charles Norris. *Treasurer:* John Reynell.

†"It is a moist piece of ground, adjoining the brick-yards where there are ponds of standing water, and therefore must be unhealthy, and more fit for a burying-place (to which part of it is already applied) than for any other service; besides, it is part of a square allotted by the late honorable proprietary for public uses, as the old maps of the city will show; our fellow-citizens would tax us with injustice to them, if we should accept of this lot by a grant from our present proprietaries, in such terms as would seem to imply our assenting to their having a right to the remainder of the square." Well argued and clearly expressed, most worthy managers!

(February 10th, 1752,) hired the most convenient house that could be procured, with gardens, etc., in Market Street near Fifth, and agreed with a matron to govern the family and nurse the sick, and provided beds and other necessary furniture. From that time "the physicians and surgeons, with a committee of the managers, have constantly and cheerfully given attendance at the house twice a week, to visit the sick, examine cases, admit and discharge patients, etc., besides daily attendance of the former." On the 7th of May, 1752, there was a new choice of managers, and a treasurer, (Charles Norris.) The managers met soon after and chose six physicians and surgeons for the ensuing year, viz., Doctors Lloyd Zachary, Thomas Bond, Phineas Bond, Thomas Cadwallader, Samuel Preston Moore, and John Redman. These gentlemen charitably supplied the medicines at their own expense till December, 1752. They are also represented to "have made their visits with greater assiduity and constancy than is sometimes used to their richer patients."

At the latter end of the year 1754, a lot of ground with a south front of 396 feet was purchased of Parker & Hinton, for the sum of five hundred pounds currency. An acre on the north side, belonging to the proprietaries, was afterwards given by them, so that possession was obtained of the entire square between Pine and Spruce and Eighth and Ninth Streets, on a part of which the hospital was afterwards erected. The foundation was laid on the 28th of May, 1755, in the presence of all the managers and physicians and of several contributors. On the twenty-seventh of December, in the following year, "the managers held their first meeting for inspecting the business of the hospital, in the room set apart for the purpose." The patients had been removed into the new building at an earlier day in this month. The record* from which we have borrowed ends with the year 1761, at which time two changes had occurred in the medical staff of the hospital by the resignation of Dr. Lloyd Zachary, in 1753, on account of disability from paralysis, and of Dr. Samuel Preston Moore, in 1759, owing to the pressure of other business; and the appointment of Dr. Thomas Cadwallader in place of the former, and of Dr. Cadwalader Evans in place of the latter.

While the records from which we have drawn show that Philadelphia was well provided with medical men of a high professional standard, it

* Our copy of this history is a reprint in 1817, from the first edition written by Franklin himself. Historical notices of the hospital have been written by Dr. C. B. Coates, and Mr. Malin, the steward of the institution; and with all desired fullness by Dr. George B. Wood, in his Address at the Centennial Celebration of the Founding of the Hospital, in 1855.

does not follow that equally satisfactory exhibitions in this way could have been made in the interior towns and throughout the Colony of Pennsylvania generally; nor, indeed, that with the increase of population in the capital itself there would be a corresponding addition of the right kind to the medical corps. It was hardly to be expected that young men academically educated would come forward, in adequate numbers and in due order of succession, to undergo a long probationary period of study at home, and then to encounter the danger and hardships of a voyage across the Atlantic, which was very different at that time to what it is now in this age of steam and scientific navigation, and in another and a foreign land to expend time and money and submit to social privations, in order to prepare themselves for obtaining the honors of the doctorate and for taking rank among enlightened and trustworthy practitioners of medicine. Fears on this head must have been felt by more than one of the accomplished physicians at the period of which we are now speaking, and suggested on their part a benevolent wish, perhaps not entirely unmixed with some natural longings of personal ambition, to provide means for carrying out a liberal curriculum of medical study at home. Without some provision of this nature, there would soon have ensued a defective supply of well-educated physicians, a lowering of the standard of the requisite attainments, abuses in the practice of medicine, and, as an unavoidable and lamentable consequence, great injury to the public health, as well by an ignorance of the prevention as of the means of the cure of diseases. The founding of a hospital, and the facilities which it gave for clinical instruction in its wards, must have imparted a degree of fixedness to what might otherwise have been but passing thoughts on the subject. Be this as it may, history tells the fact, that the foundation of the first medical school in North America was laid in Philadelphia; and that from the date of that event down to the present day, the lead thus taken has always been maintained. This assertion will not seem presumptuous if we advert to the annual concourse of students, which exceeds that of any other city on the continent. Corresponding prominence and reputation have been won for the medical literature of Philadelphia, which, counting all that emanates from it, as producer and distributor, is richer and more various than that of all other places, taken collectively, in the Union. Were we to attempt to trace, in due order of sequence, all the causes of these remarkable results, we might not be entirely successful; but we need have no hesitation in ascribing much, very much, to the character of the pioneers in the path of progress and improvement. Who were these men, is a question which will naturally be asked with some eagerness by our readers, whose wish on this occasion may fairly represent that of the profession at large. We shall proceed, without further comment, to gratify, to some extent at least, this laudable curiosity.

The men who planned the work of founding a medical school in Philadelphia, and they who joined the founders in giving it due proportions and extended usefulness, were noted for their learning and attainments, the result of their early studies at home, and of their subsequent studies and travels in different countries in Europe. They were not moved to the undertaking by the impulse of youthful vanity, to gain the adventitious distinction of mere title, nor by a desire to anticipate the slow income from regular professional labor. Imbued with the precepts and doctrines of the master-minds of the age, and possessed, through them, of the accumulated medical experience of twice ten centuries, they were fully alive to the importance of their new vocation and the magnitude of the trust confided in them when they obtained the honors of the doctorate. In a spirit of benevolence and patriotism, they determined, by associating themselves together in a corporate community, to transmit to that portion of their younger countrymen, who might hereafter wish to engage in the study of medicine, the advantages of systematic instruction in its several branches at home which they themselves had enjoyed abroad.

The leaders in this educational enterprise were Dr. William Shippen and Dr. John Morgan, both of them natives of Philadelphia, and both born, we believe, in the same year, 1735. Shippen was doubly blessed, first in the inheritance of an amiable and benevolent disposition from his father, Dr. William Shippen, Sen., and next in the example continually before him of purity of life and discharge of the Christian virtues by the same kind parent. He was early distinguished for his pleasing elocution, so much so that he was appointed to deliver the valedictory oration, in Latin, at the commencement held in the College of Newark, New Jersey, in which he had been prosecuting his studies. On this occasion he was publicly addressed by the celebrated Whitefield, who happened to be present, and who, after declaring that he had never heard better speaking, urged the young collegian to devote himself to the ministry. After a novitiate of three years' study under the direction of his father, Shippen, at the age of twenty-one years, embarked for Europe. He first went to London, where he remained some time in the family of John Hunter, while attending the lectures of Dr. William Hunter on anatomy and midwifery. In both matter and manner, he here found examples of the very highest standard, and which, it has been intimated, was not a little influential when making his own prelections and demonstrations in after-years. He was at this time an associate of William Hewson, afterwards eminent for his observations and experiments on the blood and other physiological subjects. It is not long since the son, Dr. William Hewson, alike estimable for his surgical skill and attainments and his personal and social character, has ceased to be among us. Shippen was fortunate in obtaining the friendship of the distinguished army physician, Sir John Pringle, and of Dr. John Fothergill. The latter must always be gratefully remembered by the inhabitants of Philadelphia, for his liberal gifts of anatomical drawings, valued at the time at upwards of two hundred guineas, and also of money, as contributions to the Pennsylvania Hospital, which had been erected a short time previously. Fothergill, in addition to his contributions to medical literature, was distinguished for his benevolence, his large practice and large income, and his fondness for natural history. Shippen went from London to Edinburgh, where, after devoting a proper period to the requisite studies, and attending the lectures of Cullen, Monro, and others, he took his degree.* Thence he visited France, as the physician of a lady laboring under consumption. At Paris, he made the acquaintance of Senac, whose treatises on fever and on diseases of the heart are familiar to the medical reader.

On his return to Philadelphia, after an absence of five years occupied in study and travel, in addition to the three years of preliminary study, Dr. Shippen resolved to teach anatomy on the human subject and to practice midwifery, in imitation of his eminent London preceptor, Dr. William Hunter. The first was a new and a bold undertaking, which required not only anatomical knowledge, but, also, the greatest discretion and address to obviate public prejudice and to prevent popular clamor. Happily for the cause of science, he was eminently fitted, both by natural gifts and acquired knowledge, to meet all the requirements of the occasion. Inheriting his father's unalterable good humor and popularity, graceful in person, and with manners the most conciliating, Dr. Shippen succeeded not only in disarming opposition, but in securing support out of doors for his undertaking. With these qualifications, it was an easy task for him, in the lecture-room, to impart additional interest to his demonstrations of organic structure by his facile speech and pleasing elocution. He arrived in the month of May, 1762; and soon after came the drawings presented by Fothergill, which had been committed to Shippen's care. His introductory lecture was delivered in the autumn of the same year, in one of the rooms of the State House, in the presence of many of the most intelligent citizens of Philadelphia.† His

---

* The subject of his thesis was, *De Placentæ cum Utero Nexu.*

† The first regular lectures on anatomy, on this continent, were delivered by Dr. William Hunter at Newport, Rhode Island, in the years 1754–55–56. Advertisefirst class consisted of but twelve students; and yet from this small beginning arose the medical school which, ere many years had passed, became deservedly celebrated, and in whose halls hundreds were annually congregated to hear its professors hold forth on their respective branches. Dr. Shippen lived to see and enjoy this result. The class in attendance, in the last year in which he lectured in the University, 1807, that preceding his death, numbered nearly four hundred. Three courses of private lectures were given by Dr. Shippen, unconnected with any institution; but he was henceforth to be strengthened in his career of usefulness by being associated in corporate relations with Dr. John Morgan.

Dr. Morgan had arranged with Dr. Shippen a plan for establishing a medical school in Philadelphia, and certainly no man was better fitted to contribute his full share toward securing the success of such an enterprise. Young Morgan, in 1757, took the first honors in the Philadelphia College, a literary institution which, though previously in existence as an academy, had only received its present title and corporate character two years previously. He had begun, however, the study of medicine, under Dr. John Redman, before this time, and consequently in the last years of his collegiate course. During one year of his apprenticeship, Morgan served as apothecary in the Pennsylvania Hospital. His preparatory medical studies having been gone through, he entered the provincial army in the double office of a lieutenant and a surgeon—a soldier militant and a soldier sanitary. It would seem that he acted only in the latter capacity, in which he acquired both knowledge and reputation. But it was not in Morgan's nature to content himself with mediocrity, or the limited sphere of usefulness of a provincial army surgeon. In 1760, having resigned his commission, he embarked for Europe, with a view of prosecuting his medical studies on a more extended scale, and with appliances which were not to be found at home. He first attended the lectures and operations of Dr. William Hunter, whose star was then in the ascendant in London; and whose manners and address contrasted so favorably with the rough exterior and blunt speech of his immediate precursor, Smellie. Morgan went from London to Edinburgh, where he passed two years in study and in attendance on the lectures of Monro, Cullen, Rutherford, Whytt, and Hope, in the University of Edinburgh, from whom he received his diploma of doctor of medicine in 1762, after having defended an elaborate thesis on "Pus." He now directed

---

ments of these lectures may be seen, says Thacher, in the Boston papers of that day. Dr. Hunter was a native of Scotland, and nearly related to the brothers, Dr. William Hunter and John Hunter. He was well versed in the literature of his profession, and acquired eminence both in the practice of medicine and of surgery.

his steps to the continent, and made Paris his first stopping place; here he became a pupil, for a twelvemonth, of Sue, who then occupied the foremost grade in anatomy, and who was the grandfather of the novelist Sue. The successful preparation of a kidney, by injection, with great minuteness and finish, won for Dr. Morgan a membership in the Royal Academy of Surgery in Paris, by which he became the associate of Morand, of Antoine Petit, and of Antoine Louis, its perpetual secretary, all three surgical celebrities at that time. He visited Holland and Italy, conversed with Voltaire at Ferney, and had the honor of a long conference with Morgagni, then in the eightieth year of his age. The celebrated professor of Pavia was so much pleased with the young American, that he claimed kindred with him on the strength of some resemblance in their names, and he actually gave a copy of his works with an inscription in one of the volumes: "*Affini suo præclarissimo Johanni Morgan donat auctor.*" Gratifying evidence of the esteem with which the student and traveler was regarded, on his return to Great Britain, is found in the fact of his being elected a member of the Royal Society, and admitted a licentiate of the College of Physicians in London and a member of the College of Physicians of Edinburgh.

We can readily conceive with what pleasure and pride Dr. Morgan's return would be hailed by his fellow-citizens of Philadelphia, who could not have been strangers to the manner in which he had passed his time abroad, nor to the rich stores of medical knowledge which he was accumulating, not less for their benefit than for his own reputation and usefulness. Immediately after his return, in 1765, he was elected professor of the Theory and Practice of Medicine in the College of Philadelphia,—the first appointment of the kind ever made in Pennsylvania, probably the first in the then British Provinces of North America. But this office was merely titular, since it imposed on the professor no immediate duties nor promised immediate advantage to others. Dr. Morgan felt that something more than this was due, both to himself and to his fellow-citizens; and believing that the time had come to establish a regular medical school, he took the proper measures to enlist the support of others in his plan. The trustees were induced to act as they did by a written communication from Dr. Morgan, setting forth his plan for opening a medical school under the patronage and government of the college, and by written opinions and recommendations to the same purport which he had procured from Thomas Penn, the proprietary of Pennsylvania, and Mr. Hamilton and Mr. Peters, former presidents of the board of trustees of the College. The "Discourse," in favor of the measure, was delivered by Dr. Morgan, at a public anniversary commencement held in the College of Philadelphia, May 30 and 31, 1765, so soon as he had been elected professor: it was addressed to the trustees of the college and to the citizens of Philadelphia. The speaker, after enumerating and defining the various branches of knowledge which compose the science of medicine, the order in which they ought to be studied and the pre-requisites before beginning, proceeds to argue in favor of establishing medical schools on this continent, and the tendency of and the circumstances favorable to such an undertaking. He mentions the eminent practitioners in Philadelphia, the practice of the city hospital—founded, as we have described, ten years before—the flourishing state of literature in the college, the favorable situation and populous condition of Philadelphia,* and the increase of the colonies. He next points out the advantages to be expected from the proposed institution by different parties, viz., to students of medicine, to the college, to the City of Philadelphia, and finally to the neighboring colonies. Every hope, every expectation and promise, held out by the new professor in this discourse, have been as fully realized as if they had been uttered in the spirit of prophecy. Speaking for himself, he announces his intention of giving, in a few months, a course of lectures on materia medica, and on chemistry, so far as it is allied to the former branch. "Next year I design," he continues, "to attempt a course of lectures upon the institutes and theory of medicine, which will be illustrated with practical observations." How fully and how soon his anticipations, expressed in the following sentence, were fulfilled! "Possibly, in a few years more, persons duly qualified may offer to undertake full and competent courses of every branch of medicine, and a plan may be adopted conformable to that which is followed in the so justly celebrated school of Edinburgh." He then appeals to the ambition and desire of youthful students to promote the public weal, so that they may qualify themselves, by steady application and by foreign travel, for teaching "the most difficult and important part of their profession." Dr. Morgan seems to have chosen the opportune time, and to have evoked from some of his contemporaries the right spirit, and one responsive to that by which he was himself animated. Apparently in anticipation of his appeal, the youthful Kuhn was at this very time abroad on his travels through Europe. Among those who listened to the discourse was, doubtless, young Rush, who, we may readily suppose, must have felt his heart throb with ambitious longings for an elevation to which he was in a manner invited by the laudable desire of the new professor to procure suitable associates in his undertaking.

Dr. Morgan adverted to Dr. Shippen's lectures on anatomy, and the

* The population of the city at this time may have amounted to forty thousand persons.

ability of his friend to be a professor, if the college should create a chair of this branch. Dr. Shippen, in the introductory to his first course, had proposed some hints of a plan for giving medical lectures; but the merit of recommending a collegiate system of medical instruction belongs to Dr. Morgan. He was the first professor appointed, although Dr. Shippen was the first lecturer. The former showed the feasibleness of the plan, and volunteered his services to carry it on; the latter removed the greatest obstacle to its success, and gave practical evidence in its favor. Dr. Shippen was appointed professor of anatomy and surgery in September, 1765. These two highly educated and accomplished gentlemen, imbued not only with the knowledge taught in the best medical schools of Europe, but also cognizant of the most approved methods of teaching, are now associated together as part of a regular collegiate faculty of medicine. But, with all their talents and industry, they could not, of themselves, teach all the branches recognized to be useful and necessary for a full course of medical instruction. They were not long, however, in being reinforced by men of mark and merit, whose minds had been matured by long study and travel in foreign lands.

First among these true notabilities, in the order of time, was Dr. Adam Kuhn, who was born in Germantown, now a part of the incorporated City of Philadelphia, on the 17th of November, 1741. After having enjoyed the benefits of a good academical education, which included an acquaintance with the classics, he entered the office of his father, Dr. Adam Smith Kuhn, of Lancaster, himself liberally educated, and a physician of ability. Young Kuhn set out on his travels in Europe with a view of engaging in a more enlarged and comprehensive course of study, and of gaining hospital experience greater than he could obtain at home. He first went to Upsal, at whose celebrated university he attended lectures on different branches of medicine during two years and a half, while enjoying, at the same time, the society and instructions of Linnæus, then in the height of his fame. In a letter of this eminent man to the elder Kuhn, he speaks in the highest terms of the amiable and uniformly correct deportment and devotion to study of the young American who had been confided to his charge. We next hear of Kuhn at Edinburgh, where, after defending a thesis, "*De Lavatione Frigida,*" he took his degree of doctor of medicine, in 1767. He had in the mean time traveled in Holland, France, and Germany. Dr. Kuhn, on his return in the month of January, 1768, was at once appointed to the chair of materia medica and botany in the College of Philadelphia—a strong evidence this of his professional attainments and moral worth. The young professor began to discharge, in part, the duties of his chair in the May following, by delivering a course of lectures on botany. From this date to the year 1797, or during a period of nearly thirty years, Dr. Kuhn continued to be a teacher; first in the medical department of the Philadelphia College, and afterwards, or from 1780, in the University of Pennsylvania, in which institution he was professor of the theory and practice of medicine. After the resigning of his chair, in 1797, Dr. Kuhn gave himself up entirely to the practice of his profession, in which he had acquired and continued to retain great eminence, due not only to his skill, but to his punctilious attention to the feelings of his patients and the rights of his medical brethren. He was physician to the Pennsylvania Hospital for a period of more than twenty-two years, and he succeeded Dr. Shippen as president of the College of Physicians, in the establishment of which institution by charter, in 1787, he had taken an active part. It may surprise some of our younger medical readers, who cannot walk half a mile, to learn that, notwithstanding the tax on his time and strength in his attendance on a large circle of patients, Dr. Kuhn only used his carriage in inclement weather. But then he was regular, even to abstemiousness, in his diet, and by this means he reached the good old age of seventy-three years. His death took place in July, 1817.

Nearly contemporaneous with the appointment of Dr. Kuhn to the chair of materia medica and botany, was that of Dr. Thomas Bond to the professorship of clinical medicine. Of the professional and literary merits of this gentleman we had occasion to speak in enumerating the medical worthies of Philadelphia in the first half of the eighteenth century. The first medical fruits gathered from the establishment of the hospital were those growing out of the clinical lectures (in 1766) given by Dr. Bond, anterior even to his appointment in the college. He may be regarded as the Nestor of the medical faculty of that day, the other members of which he had seen grow up from childhood. Notwithstanding the delicacy of his constitution and the predisposition, in early life, to pulmonary consumption, Dr. Bond reached the advanced age of seventy-two years—a result attributable, in a great measure, to the regularity of his habits of living, especially to his rigid attention to diet, and guarding against the effects of changes of temperature.

There yet remains one name, the greatest among the great, that of Rush, to complete our notice of the first medical faculty attached to the Philadelphia College. Benjamin Rush was sent to the academy at Nottingham, where Morgan and Shippen had been at an earlier date, and afterwards to the college at Princeton, in which he took the degree of bachelor of arts. His preliminary medical studies were begun, and continued during a period of six years, in the office of Dr. Redman, which was a nursery of professors. Among the first books read by the young student were the writings of Hippocrates; and one of his earliest self-imposed tasks, at this time, was a translation of the Aphorisms of the Coan sage. He was one of the first small class who attended the anatomical lectures of Dr. Shippen, in 1762. On the completion of the period of office study, which he never complained of as being too long, Rush visited Europe; and having complied with the requirements of its university, took the degree of doctor of medicine in Edinburgh, in 1768. The subject of his inaugural thesis was, "*De Coctione Ciborum in Ventriculo.*" The following winter was passed in London, and the spring in Paris. His return to Philadelphia, in the autumn of 1769, was signalized by his appointment to the chair of chemistry; thus making him the associate of Morgan, Shippen, Bond, and Kuhn, in the newly organized medical department of the Philadelphia College. This organization was maintained until the war of Independence; and it was then affected less by the derangement incidental to a revolution than by the founding of the University of Pennsylvania—at first a rival and antagonistic institution, but eventually the successor to the Philadelphia College, and inheritor of its medical faculty.

In the year (1768) immediately preceding that in which the last professor in the medical department of the Philadelphia College, Dr. Rush, was appointed, the first medical commencement was held. On this occasion the degree of bachelor of medicine was conferred on ten gentlemen, after suitable examination of their fitness to undertake the practice of their profession. At first this was the only degree granted, and for a while afterwards both it and the doctorate were conferred on candidates according to the extent of the qualifications and previous study of each of them respectively. A further change was subsequently effected, in the title of doctor of medicine being the only one granted.

Here we would pause in our narrative, to contemplate the broad foundation laid for medical education on this continent by its founders, and the high standard of attainments which they required of those who aspired to enter the ranks of the profession under their guidance and that of their successors. It was part of the good fortune of the new medical school in Philadelphia that it was planted in an atmosphere of professional learning, and its growth watched and fostered by men of the like feelings and aspirations with the first cultivators. Under other and less favorable auspices and direction, and left to the uncertain impulses and vagaries of popular belief and prejudices, there might have sprung up a brood of "Indian doctors," quack salvers and medicasters, proclaiming, with all the fervor of mock patriotism, the superior adaptation of the American system, as they would have called it, to the constitution of the inhabitants, and to the treatment of the diseases of this climate. It is worthy of remark, that the most eminent practitioners of medicine in Philadelphia, during the latter half of the last century, were good scholars—men of learning, and actively participating in, when they did not originate, the various plans set on foot for general and medical education and the advancement of literature and science. The usefulness, the dignity, and the success of the first medical school rested on the broad platform of both ancient and contemporary learning laid by its founders, and who, unless they had been early imbued with this learning and had submitted to a long and thorough training, would not, in the first instance, have felt the necessity of the work, nor, subsequently, have had sufficient confidence in themselves to carry it on to completion. Drs. Morgan and Shippen, and their associates whom we have named, enforced with more than the eloquence of words the correctness of the standard which they were about to offer for the edification and eumulous imitation of the American youths who should come after them. They could point to the examples of long and patient study and travel furnished in their own novitiates. They did not feel the eager desire, the hot haste, so characteristic of the young men of the present day, to run through, in the shortest possible period, a course of studies which shall allow of their procuring a degree and implied license to practice physic. Time, these founders were well aware, is an important and an indispensable condition for acquiring knowledge, and without it genius alone will be more apt to mislead than to benefit either its possessor or mankind in general. "It is now more than fifteen years," wrote Dr. Morgan, in the preface of his Discourse, "since I began the study of medicine in this city, which I have prosecuted ever since without interruption." During the first six years of this period, as we had occasion to state, he served, according to the custom of the time, an apprenticeship with Dr. Redman, himself in a large practice, and, as before mentioned, a man of learning, and a graduate of Leyden. The next four years were passed as surgeon in a provincial regiment, in the war then raging between England and France. Continuing his account of his medical studies, he tells his readers: "The last years I have spent in Europe, under the most celebrated masters in every branch of medicine, and spared no labor or expense to store my mind with an extensive acquaintance in every science that related, in any way, to the duty of a physician, having expended in the pursuit a sum of money of which the very interest would prove no contemptible income." Dr. Shippen, as we have seen, after having received full academical and collegiate instruction, studied medicine in his father's office for three years, and spent three more in study and travel in Europe. Dr. Kuhn, as the reader has also learned, was well grounded in classical learning, went through his preliminary studies in medicine with his father, and then studied and traveled in Europe for seven years. Dr. Rush was a student under Dr. Redman for six years and passed three more abroad, making in all nine years given to the study of medicine before he asked for a place among those who practiced it.

In his advocacy of a high standard of preparatory attainments for the study of medicine, and while insisting on its relations to every other science, Dr. Morgan makes no attempt to compromise with vulgar prejudices, nor with ignorance or indolence, in order to win popular favor for the cause which he has so much at heart. He looks with confidence to the transplanting of medical science "into the Philadelphia College, and for the improvement of every branch of the healing art." Nothing less than the standard recognized at Edinburgh, at Leyden, and at Paris and Montpelier, seemed worthy of being adopted in Philadelphia. The transplantation was made, the tree flourished, and its fruit has been ever since distributed over the land, in addition to the increase of similar growths by ingrafting from the parent stem. All honor, then, to Dr. John Morgan and Dr. William Shippen! Statues have been erected to men and celebrations kept in their honor, who have not deserved as well of the people of the United States as the illustrious founders of the first medical school on this continent.* Their names must always be held in veneration, not only by every successive faculty and class of alumni of the University of Pennsylvania, but by those of all the medical colleges in the Union, which derive or profess to derive nutriment and strength from the same wide and fertile fields of medical science and of medical ethics.

The regular sessions of the medical school could not fail to be interrupted during the revolutionary war, and especially at the time that

* "In 1768, a medical school was organized under the direction and government of the College of the Province of New York, then called King's College, and a board of professors appointed to teach the several branches of medical science. The instructors in this early school were, Samuel Clossey, M.D., Professor of Anatomy; John Jones, M.D., Professor of Surgery; Peter Middleton, M.D., Professor of Physiology and Pathology; James Smith, M.D., Professor of Chemistry and Materia Medica; John V. B. Tennent, M.D., Professor of Midwifery; and Samuel Bard, M.D., Professor of the Theory and Practice of Physic. On all these branches lectures were regularly given; and the degrees of Bachelor of Medicine and Doctor of Medicine were conferred by the college."—*Dr. Hosack's Inaugural Discourse at the Opening of Rutgers Medical College in the City of New York*, November 6th, 1826. In 1769, we are told, by the same authority, that the first-mentioned of these degrees was conferred upon Samuel Kissam and Robert Tucker. "In 1770 the degree of Doctor of Medicine was conferred upon the last-mentioned physician, and in May of the succeeding year the same degree was conferred upon the former." The establishment of a hospital in New York was at a somewhat subsequent date.

Philadelphia was occupied by the British troops. Three of the professors were appointed to office in the American army. Dr. Morgan held for two years, or from 1775 to 1777, the post of "Director-general and Physician-in-chief to the General Hospital," and used his utmost endeavors, but with imperfect success, to establish system in his department. Complaints, it should rather be said clamors, without adequate cause, were raised against him; but their injustice, fully ascertained in the end and so admitted by Congress, did not prevent that body from removing him from office. Dr. Shippen, in 1776, took a place in the medical department of the army, which he continued to hold until the year 1780; but without his allowing it to interrupt his lectures, except during the winters of 1776 and 1777. Dr. Rush served in the capacity of physician-general to the middle department in the revolutionary army. The observations made on the diseases of hospitals and camps during his period of service were turned to excellent account, in after-years, in his lectures on the theory and practice of physic. But a still more signal honor will ever be associated with the name of Benjamin Rush, as one of the immortal band of the signers of the Declaration of Independence.

Before resuming the thread of our narrative of the progress of medical teaching in Philadelphia, we would invite the attention of our readers to the galaxy of distinguished men in all the departments of medicine during the decade between 1760 and 1770, that in which the two founders of the school, Drs. Morgan and Shippen, and their first associates, Drs. Kuhn and Rush, traveled and attended lectures and hospitals in Europe. The period was a remarkable one, and beyond what it would seem to be even to those who have paid some attention to medical biography. A zealous inquirer might, within the time above mentioned, have seen and spoken to the following array of eminent men, have listened to their prelections, and discoursed with them on their works. Some of them have already been mentioned, as teachers and friends of the young American students; but a repetition of their names in the present connection will not, we are sure, be considered inappropriate. Monro the first was still living when Shippen and Morgan were in Edinburgh, and he could have encouraged them in their project of founding a medical school in Philadelphia by relating the success which had attended his own efforts, in conjunction with Dr. Alston, to establish that of Edinburgh, in the early part of the century. He taught anatomy and surgery, while his associate lectured on materia medica and botany. An infirmary was then erected, in which a course of clinical lectures on surgery was opened by Dr. Munro, and on medicine by Dr. Rutherford. To these names were added those of Plummer, Whytt, and Sinclair, by which the first faculty was completed. That which succeeded, and was in activity during the period of which we are now speaking, gave at once to the Edinburgh school a station and fame second to no other in Europe. First came Cullen, who, leaving the chair of chemistry in the University of Glasgow, was elected to that in the University of Edinburgh as successor to Plummer, and then to the chair of materia medica, following Alston, and, in the year 1776, to that of the theory and practice of medicine, which had been filled by Rutherford. With Cullen were associated Monro the second, Black, the pupil and successor of Cullen in the chair of chemistry, and Hope, who took the place of the latter in that of materia medica, and finally Hamilton, as professor of midwifery. John Brown, who was to run a short career of fame and of misfortune, as first the pupil, protegé, and friend of Cullen, and subsequently the rival and bitter enemy of the great teacher, was now married and receiving medical students into his family as boarders. He was, at this time, best known for his mastery of Latin, in which language he wrote inaugural theses for candidates for graduation. The habits of intemperance, which shortened his life and ruined his fortunes, had already begun to show themselves.

In London, although there was neither university nor medical college, a visitor would meet with great teachers and eminent practitioners in anatomy, surgery, and midwifery—lecturers in hospitals, and extensively engaged in private practice. Percival Pott was surgeon to St. Bartholomew's Hospital, in the meridian of his fame as a humane and skillful operator, and a careful and watchful observer and writer. He had published his work on the "Injuries to which the Head is liable from External Violence." Sharp, the most noted pupil of the great Cheselden, and so long surgeon of Guy's Hospital, had, in a measure, withdrawn himself from business, and was traveling on the continent, to recruit his health. Dr. William Hunter, distinguished as an anatomist, physiologist, and accoucheur, with the additional merit of elucidating his subject by a happy manner of lecturing, was preparing his great work on the "Anatomy of the Human Gravid Uterus." It was begun in 1751, and not completed until 1775. He gained reputation, and access to the wealthy and the titled, which was denied to his predecessor and countryman, Smellie, who, with all his great merits as teacher and writer, was ungainly in person and even repulsive in manner. Denman, the pupil of Smellie, was now known as the author of "Essays on the Puerperal Fever and on Puerperal Convulsions;" 1768. John Hunter, the brother of William, had returned from the expedition to Belle Isle, where he acquired his knowledge of gunshot wounds, and was teaching anatomy and surgery to a class, sometimes not exceeding twenty persons. After he was elected (in 1767) a fellow of the Royal Society, he was in the habit of adjourning from the meetings of this body to some coffee-house, to discuss with others such subjects as were connected with science. The suggestion came from Hunter to Dr. George Fordyce, and Mr. Cumming, an eminent mechanic, and they were soon joined by Sir Joseph Banks, Dr. Solander, Dr. Muskelyne, the astronomer, Sir George Shuckburgh, Sir Harry Englefield, Sir Charles Blagden, Dr. Noothe, Mr. Ramsden, Mr. Watt, of steam-engine celebrity, and many others. Banks and Solander sailed in the following year with Cook on his voyage of circumvavigation. Pringle had written his work on Diseases of the Army, was knighted, and made physician to the new king, George III. Fothergill, in the midst of a large and lucrative practice, formed a complete botanic garden at his estate at Upton; and in the city he was making a valuable collection of shells and other objects of natural history. He had an artist at the Cape of Good Hope, and another to range the Alps, to aid him in these acquisitions. He was active in suggesting means for the improvement of the public health, and liberal in his benefactions. George Fordyce was teaching chemistry, materia medica, therapeutics, and pathology, and in this period he had issued the first part of his Elements of the Practice of Physic. Heberden, now in large practice, was accumulating that experience which he transmitted to his brethren in his "Commentaries." Hillary had returned from a six years' residence at Barbadoes, and written his observations on the climate and diseases of that island. Motherby was most probably engaged on his medical dictionary, but which did not appear until the year 1778. Short was still writing on the mineral waters of England. Percival, after having obtained his degree at Leyden, and traveled in France and the Netherlands, was settled in Manchester, to the medical and literary character of which he greatly contributed by his luminous histories and observations. His code of medical ethics dates in 1800. Darwin was living at Lichfield, but his "Botanic Garden" did not appear until 1781, and the first part of his Zoonomia in 1793. But in the period of which we are speaking, two eminent English poets were living—Armstrong and Akenside; the first of whom wrote "Medical Essays," the second on Dysentery, Causus, etc.

In Dublin, McBride was writing on scurvy. His "Introduction to the Practice of Medicine" was of a later date. Cleghorn was enjoying the reputation derived from his book on the "Epidemical Diseases of Minorca," written when he was but twenty-five years of age. He had become at this time a lecturer on anatomy. Rutter had sent out in two large volumes his "Synopsis of Mineral Waters." His "*Materia Medica Antiqua et Nova,*" etc., published in the next decade, had to encounter the searching criticisms of Cullen.

Paris, during this period, contained many illustrious men in the different walks of professional life. Antoine Louis had just succeeded Morand as secretary of the Royal Academy of Surgery, to which body he imparted new life and action by his own contributions, collections of memoirs, and his famous "Eloges." He was at the same time the learned expounder of difficult questions in legal medicine. His rival, Antoine Petit, celebrated for his surgical tact and sure prognosis, had the additional merit of being the preceptor of Desault, who was beginning to show himself the able and attractive, though not eloquent, lecturer on surgery, before he had acquired the title of professor. Sabatier had written his anatomy, and was engaged in the preparation of his celebrated work on operative surgery. Le Dran was living, at an advanced age, with the reputation of a successful surgeon and judicious writer. Rouen could still show her eminent surgeon in the person of Le Cat. Levret had acquired a wide-world reputation as a teacher and practitioner of midwifery at this time, and had issued the third edition of his work on the Art of Accouchments. Bordeu, in a series of dissertations, had shown the therapeutic value of mineral waters in chronic diseases; had written learnedly on the pulse; and he was now promulgating his Investigations into the Mucous Tissue, which formed part of the groundwork of the brilliant structure raised in after-years by Bichat, in his "General Anatomy." Senac lived to the close of this decade, (1770.) As a physician to the king, Louis XV., he chanced to enjoy a vogue at court which was trivial in duration and weight compared to the fame acquired by his work on the Structure and Diseases of the Heart,—one of the most remarkable of the century. Lieutaud, introduced by Senac to the same circle, had written his anatomical essays, his Practice of Medicine, and Materia Medica, and the still more known production on morbid anatomy, (*Historia Anatomica-Medica.*) Quesnay, attached to the court as physician in ordinary to Louis XV., and perpetual secretary to the Royal Academy of Surgery, was better and more advantageously known as chief of the economical school, so called. He wrote, at this period, a treatise on Physiocrasy, or the Government most advantageous to the Human Race. Tronchin, of Geneva, the amiable, the good, unspoiled by fashion and the flattery of the women, was then the leading practitioner in Paris, with the appointment of physician to the Duke of Orleans. Lorry, a prolific writer on most of the branches of medicine, is chiefly known to us for his treatises on Melancholy and the Diseases of the Skin. Daran was living, with the reputation of great surgical talent, and especially for his success in healing strictures of the urethra by bougies; he was praised for his humanity to the sick during the plague in Messina. But these fine traits of character were disfigured by his quackery, in keeping the composition and use of his bougie and auxiliary means of treatment a secret. Sue, (Jean Joseph,) the preceptor for a while of Morgan, was one of the professional men of mark in Paris at this epoch, as an anatomist, and associate chief surgeon of the Charité Hospital. His dissections of the human body, while truer to nature, can hardly be considered more repulsive than the dissections of the human mind by his grandson, if we read right, Eugene Sue, the novelist of the present day.

The school of Montpelier possessed at this time Sauvages, so celebrated ever since for his *Pathologia Methodica* and *Nosologia Methodica*, by which he gained the foremost rank as a nosologist. Barthez had just been appointed to a chair, and began to develop the subtleties of his incomprehensible vitalism: he showed his ingenuity by making it square with the richest therapeutical applications. Venel had become professor of materia medica, and he must be regarded as the first to introduce chemistry to its proper place in the faculty of the Montpelier medical school. In conjunction with Bayen he had made analyses of many of the mineral waters of France, and by undertaking those of Seltz he prepared the way for the manufacture of artificial gaseous waters. Pomme wrote his large treatise on the Vaporous Affections of Women, and claimed for the use of water, internally and externally, virtues approximating to a panacea. Fouquet, an unsuccessful competitor, at forty years of age, for the chair of Sauvages, by "*concours*," gained the object of his ambition at another trial, when he was upwards of sixty, by succeeding Grimaud. His remarkable treatise on the Pulse appeared in 1767. In this work he attempts to show that each organ in disease has its characteristic pulse, and that therapeutical agents, such as opium and blisters, have each their peculiar manifestations in this way. His theses and memoirs are numerous, and embrace a great variety of topics. Astruc was still living in the first half of this decade, to discourse on the venereal disease and the diseases of women, on both of which subjects he had written such masterly treatises.

Buffon had issued fourteen of the thirty-six volumes of his great work on Natural History, in which philosophy and science were clothed in the rich dress of eloquence. Accuracy in the detail of animal structure was insured by the minute observations and dissections of his friend and collaborator, Daubenton. Bonnet, of Geneva, was now known for his "Contemplations of Nature," and he exhibited his talents as a naturalist and metaphysician, with the possession, at the same time, of a practical ability, which caused him to be elected a member of the great council of state. His speculations on the co-ordination of all created beings and the indefinite pre-existence of germs, have furnished themes for innumerable commentaries. Geoffroy was yielding all the time he could snatch from the cares of a large practice to comparative anatomy and zoology. He gave utterance also to the precepts of hygiene in pure Latinity. Antoine Jussieu was no longer living; but his brother Bernard was now actively engaged in making a systematic division—the natural method, as it has been called—of the vegetable kingdom, and earning the eulogy bestowed upon him by Cuvier, of being "the inspiring genius of modern botanists."

Italy, the nurse of modern medicine as she has ever been, since, an active contributor to its progress, could furnish in this decade the classical names of Morgagni, Spallanzani, Fontana, Caldani, and Galvani. The great work of the first named, *De Sedibus et Causis Morborum per Anatomen Indigatis,* appeared in 1762, when its illustrious author was in the eightieth year of his age. Spallanzani, though not a physician, takes a leading place among experimental physiologists. He had written in this period, on generation, and the action of the heart; and described a mountain journey, with observations on the origin of springs. Fontana sent forth dissertations on irritability and various other physiological questions. His treatise on poisons did not appear until 1781. Caldani, the pupil and afterwards the successor of Morgagni, at Padua, was also long the correspondent of Haller, whose experiments he repeated. He wrote largely and experimented on anatomy, physiology, and pathology. Galvani was professor of anatomy in Bologna in 1762, but had not yet made those discoveries and experiments in animal electricity which have made his name ever famous.

In the north of Europe, the University of Upsal was now celebrated for its professors, among whom shone, with peculiar lustre, the names of Linnæus and Bergman. Of the first, as the great naturalist of the eighteenth century, and more especially eminent in botany, it is unnecessary to speak here. His merits as a writer on materia medica are recognized. Dr. Kuhn, we have seen, was a student of the celebrated Swede. Bergman has the merit of being a discoverer in chemistry; as, for instance, of oxalic acid and the acid character of what was then called fixed air: he systematized mineralogy, and was the first to analyze mineral waters after a methodical and critical manner.

Gaubius, the successor of Boerhaave in the school of Leyden, then ranking as the first in Europe, had not long before (1758) issued his Institutes of Medical Pathology. His "Adversaria" was of a later date. In the number of his associates was Albinus the second,* (Bernard

* The first Albinus, whose real name was Weiss, (White,) was also a professor in Leyden, and the father of the great Albinus, and of Christian Bernard, whose reputation was inferior only to that of Siegfried.

Siegfried,) who, as anatomist and teacher of anatomy, was almost without a peer in the eighteenth century. His researches in this branch, and in physiology, zoology, and botany, under the title of *Annatotiones Academicæ,* were published in successive fasciculi, the last of which appeared in 1768. Camper was another name of which Holland had reason to be proud at this time. After having taught anatomy, surgery, and medicine, in the Athenæum of Amsterdam, he had been transferred to a chair of surgery, anatomy, and medicine, in the University of Groningen, and was sending out, almost annually, a dissertation on comparative anatomy or physiology, and, occasionally, on medical jurisprudence.

The University of Gottingen, founded by George II., was rising rapidly to eminence, and already rivaled the oldest institutions of learning, under the presidency of the great Haller, whose name alone would have attracted students to its halls from all parts of Europe. An enlarged edition of his great work, under the modest title of First Lines of Physiology, (*Primæ Lineæ Physiologiæ,* etc.,) appeared at this time, (1765,) and thenceforward this branch rested on a demonstrative and scientific basis. His knowledge of anatomy and botany would, in either of the two, have conferred distinction on other men; while as a poet he could take a place among the most favored devotees of the muses, and thus shine in a twofold light, as the follower of Apollo. He surrounded himself with associates, brought to Gottingen at his recommendation, who were eminent in the different branches of medicine. There were Vogel, (Rudolphe Augustin,) teaching materia medica, Roederer, obstetrics, the two Richters, and George Gottlob and his more celebrated nephew, Augustus Gottlob, surgery.

The same services that were rendered by Haller to the University of Gottingen were performed by Van Swieten for the medical institutions of Austria, which, under the encouragement offered by the Empress, Maria Theresa, were re-organized by him on a larger and more liberal plan. Van Swieten is best known as the learned, laborious, and patient commentator of Boerhaave; and this may connect his name with that of Gaubius and of Haller, who engaged in the same labor of love. He induced De Haen to come to Vienna, and encouraged him to found the school of clinical medicine, which, under the successive direction of Stoll, J. P. Frank, Hildebrand, etc., has maintained its high character to the present day. Quarin was teacher at this time of materia medica and the institutes of medicine in Vienna. He was also the receiver of one thousand pieces of gold for his frank reply to the question of the Emperor Joseph II.: how long that prince might be expected to live. Greding, of Zwickau, was working for that eminence which he subsequently reached, by his careful study and appreciation of the best methods of treating the insane, and by his numerous and carefully conducted observations to determine the organs affected in this class of sufferers. Theden, a favorite of the great Frederick, and who had accompanied that monarch in his wars, was at this time surgeon-general of the artillery, and was laying a foundation for Prussian surgery. We must not omit, in these sketches, the name of Zimmerman, whose work on dysentery was pronounced by Cullen to be superior to all that preceded it.

Of these celebrated men whom we have named, some of them were the learned teachers, all of them the bright exemplars, in observation and experiment, and in patience of thought and prolonged study, of the founders of our first medical school. From this school have grown, at different intervals, the medical department of the University of Pennsylvania, the Jefferson Medical College, and the Pennsylvania Medical College, in the City of Philadelphia, and the numerous and flourishing schools in the Southern and Western States. The present status of our city institutions is almost entirely owing to the merits and reputation of their parent, under it must also be admitted the genial influences of situation, climate, and the character of its people. Some names have yet to be introduced in our brief historical sketch, in order to show how the Faculty, which has ever since continued to shed a lustre on American medicine, was completed. We pass over, with a simple mention, the unjust and discreditable act, in 1779, of the Pennsylvania legislature, by which the property of the Philadelphia College was sequestered, and converted into part of the endowments of the newly erected University of Pennsylvania. A tardy atonement for this wrong was made in 1789; and the college was merged in the university in 1791. The medical faculty at this last date was as follows: Dr. Shippen, *Anatomy, Surgery, and Midwifery*, with Dr. Wistar for his adjunct; Dr. Kuhn, *Theory and Practice of Medicine;* Dr. Rush, *Institutes of Medicine and Clinical Medicine;* Dr. Hutchinson, *Chemistry;* Dr. Griffitts, *Materia Medica;* and Dr. Benjamin Smith Barton, *Natural History and Botany.* We miss from this list the honored names of Morgan and Bond. Death had previously stricken them down; the first in 1780, at the early age of fifty-four years; the second in 1784, at the advanced age of seventy-four. We meet with new names in the Union Faculty, viz.: Hutchinson, Griffitts, Wistar, and Barton. The two first mentioned had but a short tenure of office, owing, in one case, to death; in the other, to resignation.

Dr. James Hutchinson was born in Bucks County, Pennsylvania, in 1752. After receiving the degree of Bachelor of Arts in the College of Philadelphia, he began the study of medicine in that city, under the direction of Dr. Evans, and continued it in London under the immediate guidance of Dr. Fothergill, already known to our readers as a benefactor to the Pennsylvania Hospital. Such was his proficiency in chemistry that the trustees of the College of Philadelphia presented him, in 1774, with a gold medal. On his return home, he entered the army, and served as physician and surgeon during the whole of the revolutionary war. In 1779 he was elected one of the trustees of the infant university; and in the year 1789 he received the appointment to the chair of materia medica and chemistry in that institution. In addition to his professorship, Dr. Hutchinson held the post of physician to the Pennsylvania Hospital, and to the port of Philadelphia. He was also a member of the Philosophical Society. To unquestionable talents and opportunities for acquiring professional distinction and enlarging his field of usefulness, he added a winning address and popular manners. The road to fame and wealth thus opened to him, was, however, suddenly closed by death from the yellow fever of 1793, while he was yet in the very strength and maturity of his days.

A longer period was given for useful and benevolent operations to his colleague, Dr. Griffitts, although it fell short of the threescore years and ten. Samuel Powell Griffitts was born in 1759, of parents of the religious Society of Friends, more commonly called Quakers, of which he always continued to be a member. He acquired an accurate knowledge of the Greek and Latin languages in the College of Philadelphia, and a mastery of the French so as to speak it with correctness and fluency. His medical studies were begun under Dr. Adam Kuhn, with whom he became, in after years, associated in the medical faculty of the university, as he was ever after by the ties of friendship. He visited Europe in 1781, and directed his steps to Paris, where he attended the lectures and hospitals, at a time when Lavoisier, Fourcroy, and Berthollet were laying the foundation of modern chemistry. After this he attended a course at Montpelier, so long celebrated for its medical school. Having traveled through the south of France, he repaired to Edinburgh, at that time the chief seat of medical science in Great Britain. There he enjoyed the instructions of Cullen, Monro the second, Gregory, Black, and Hamilton. Not long after his return home, Dr. Griffitts, following the impulses of his kindly nature, regulated at the same time by sound common sense, set about, in 1786, organizing the Philadelphia Dispensary, of which he must be regarded as the true founder. As a charity for the relief of the sick, it is only second in importance to the hospital: as a means for clinical instruction, it has not been turned to the account of which it is susceptible. For the first seven years, Dr. Griffitts served as manager to this institution; and we are told that, during a period of forty years, he visited it, with few exceptions, daily. He was one of the original members of the College of Physicians, at its establishment in 1787, and a member of the Philosophical Society when Franklin was president. He was among the earliest advocates for preparing a national pharmacopœia,—and he was in consequence appointed by the College of Physicians to prepare an essay of a pharmacopœia, which was read in the first national convention held at Washington on the first day of January, 1820.

Dr. Benjamin Smith Barton was a native of Lancaster, Pennsylvania. He visited London and Edinburgh, and prosecuted his medical studies in those cities. He afterwards went to Gottingen, where he obtained the degree of Doctor of Medicine. Blumenbach was then eminent in physiology and comparative anatomy, and was on the point of bringing out the first number of his *Collectio Craniorum*, etc.; and Osiander was beginning to take that leading stand in obstetrical teaching which he subsequently acquired. Dr. Barton was appointed, on his return from Europe, in 1789, professor of natural history and botany in the College of Philadelphia. On the union of the two institutions, in 1791, he retained this title, but without the privileges of the other professors. He succeeded, in 1796, to the chair left vacant by the resignation of Dr. Griffitts, but with the substitution of botany for pharmacy, in addition to materia medica. He held this post until the death of Dr. Rush, whom he succeeded in the chair of the theory and practice of medicine, in 1813. His tenure of office was a short one; it being terminated by death in December, 1815, in the fiftieth year of his age. Dr. Barton did much to disseminate a fondness for botany and natural history in general, and was successful in adding, both by his own observations and the investigations of his pupils, to a knowledge of the medicinal properties of numerous indigenous plants. His "Contributions toward a Materia Medica of the United States," and the inaugural theses of several of the graduates of the university, also published, are enduring evidences of his services in this way. To him also is awarded the merit of writing the first American elementary work on botany.

The name of Wistar is not only conspicuous in the annals of anatomical teaching in the University of Pennsylvania, but it is permanently affixed to the museum of which he was the founder, and to the formation of which he so largely contributed. In science he will be remembered as president of the Philosophical Society; and at the present time his name is familiar as household words, to indicate the literary and social meeting, called "Wistar Party," which finds scarcely a counterpart out of Philadelphia. Dr. Caspar Wistar was born in Philadelphia, in 1761: his parents and ancestors, on both sides, were of the Society of Friends. His academical studies made him a good scholar in Greek and Latin:

he was a slow learner, but a persevering and successful student. His choice of a profession was determined by the circumstance of his volunteering, when a boy of sixteen, to aid in ministering to the relief of the wounded, after the battle of Germantown, in 1777. He studied medicine under Dr. Redman, and afterwards surgery under Dr. Jones. In 1782, young Wistar took the degree of Bachelor of Medicine, in the College of Philadelphia. After a year spent in England, he repaired to Edinburgh, and obtained the honors of the doctorate in 1786; his inaugural dissertation being entitled "*De Animâ Demissâ.*" Previously to this event, he had been elected, for two successive years, one of the presidents of the Royal Medical Society of Edinburgh. On his return to his native city he began a career of usefulness in his profession, as a teacher and practitioner, which won for him the love of his patients and the esteem of his fellow-citizens. We need but advert to the danger to his life from an attack of yellow fever, in 1793; to his being made a physician to the Pennsylvania Hospital in the same year; to his sixteen years of valuable services rendered to that institution; to his appointment to the chair of chemistry, in the college, in 1789, and to the post of adjunct professor of anatomy, surgery and midwifery, in the university, in 1792. The year 1808, that of the death of Dr. Shippen, gave to Dr. Wistar the entire chair of anatomy and of midwifery, which was still attached to it. Surgery had been recognized as an independent branch, and the chair was filled by Dr. Physick. Dr. Wistar soon (in 1810) divested himself of midwifery, and directed all his energies to demonstrations and prelections on anatomy. As a teacher of this branch, it is no exaggeration to say, for we have had large opportunities of making comparisons, that he was unrivaled. The dry bone, in the hand of the lecturer, seemed, in the eyes of his wondering class, to become an object of beauty; and its processes and foramina as attractive as the finest outlines of form and delicate turns of the joints, chiseled by a Phidias or a Canova. By the time Wistar had got to the sphenoid bone, what with looking at the magnified model carved in wood, listening to his explanations, and then handling and closely inspecting the bone itself, one of others handed over for the purpose to sub-classes of twelve, the students felt that they were intrusted with a precious deposit, a minute acquaintance with which seemed to them to be of far greater importance than to be initiated into the mysteries of the Cabala, or even to square the circle.

The year 1805 was memorable in the history of the medical department of the University of Pennsylvania. In it Dr. Rush was formally elected to the chair of the theory and practice of medicine, although he had been performing all the duties pertaining to it for some years previously, or since the resignation of Dr. Kuhn, in 1797; and Dr. Physick was made professor of surgery, which now, for the first time, was admitted to a separate and equal rank with the other branches.

Dr. Philip Syng Physick was born in Philadelphia, in 1768. His father was an Englishman. Young Physick took the degree of Bachelor of Arts in May, 1785, and began, in the following month, the study of medicine in the office of Dr. Adam Kuhn, in which he remained for three years. In the beginning of the year 1789, he went to Europe, and became a private pupil of John Hunter, and a member of his family. In January, 1790, he was made house-surgeon to St. George's Hospital for one year; and in the following year went to Edinburgh, where he graduated in May, 1792. His thesis, "*De Apoplexiâ*," was written without the aid of any of the professional grinders who so often assist the indolent, or those unconversant with the Latin language, to perform this duty as well as to answer the questions which would be propounded. The year after his return was that of the ever-memorable pestilence of yellow fever, in 1793. By a strange mistake of his biographers, including his successor in the chair of anatomy, Dr. Horner, and his son-in-law, Dr. Randolph, he is represented to have been a resident physician at the Bush Hill Hospital, which was set apart for the reception of patients ill of the fever, during the period of the epidemic in this year. His services in this way were, in fact, rendered in the fever year of 1797. He was elected a surgeon to the Pennsylvania Hospital in 1794, and continued in this post until 1816, or a period of twenty-two years. In 1800 he began a course of private lectures on surgery, and in 1805 was called to the university as professor of this branch, which he continued to teach until the year 1819, and then only abandoned it to take the chair of anatomy. The entrance of Dr. Physick into the university was a measure not less of policy, in reference to its interests, than of gratification and honor to himself. He had opportunities now for disseminating those principles of surgery, which he had learned from his great preceptor, John Hunter, and which were strengthened and enforced by his own meditations and experience. During a period of fifteen years of public lecturing, Dr. Physick made his opinions the received canons in surgery over a greater extent of territory than it has ever yet fallen to the lot of any teacher of this branch of medicine to accomplish. In listening to him, the students felt that they were addressed by one who spoke with an authority derived not merely from office, but from an intimate knowledge of his subject and an entire conviction of the correctness of what he was saying. With him there was no superfluity of phrase, no attempt to embellish the truth. That which he knew from long experience had been his guiding star, ought, he believed, to fix in like manner the attention of others. When, as has happened within our observation, on a cloudy winter morning, he held forth to his class with his lecture in one hand and a candle in the other, the attentive and almost venerating students might believe for the moment, and especially when looking at his strongly defined features and pale face, that he was a messenger from beyond the grave (*outretombe*) who had come to announce to them great and important truths, which it behooved them to know well and to practice faithfully. Without entering into a specification of the distinctive character and merits of Dr. Physick, even in a professional point of view, we would touch on one topic, viz., his conservative surgery, that by which he showed how often a member, ruthlessly given over to amputation by mere operators, mechanics in their profession, could be saved by the slower but less showy use of ordinary therapeutical agents, aided by suitable posture and regimen.

What a glorious privilege was that enjoyed, for nearly a decennial period, by the students attending the medical lectures in the University of Pennsylvania, to pass from the room of the great teacher of anatomy, in the person of Dr. Wistar, to that of Physick, the "father of American surgery," and thence to go and hear the prelections of Rush, the American Hippocrates, and the father of American medicine, the medical philosopher, the philosophical philanthropist, and the tried patriot; learned yet ever learning, diligent in collecting facts, and ready, when the opportune moment came, to expand facts into principles; whose purity of life, from boyhood to advanced age, was a practical commentary on his elevated ethics, and whose pen and tongue were enlisted on every theme that could give value to the independence of his country, by improving the health, cultivating the mind, and preserving the morals of his fellow-citizens.

The chair of chemistry left vacant by the death of Dr. Hutchinson, in 1793, was offered to Dr. Priestley, who declined, and it was then conferred on Dr. Carson, who died without his having been able to give a lecture. Finally Dr. James Woodhouse was elected, in 1795. This gentleman was born in 1770, was a pupil of Dr. Rush, and for a short time surgeon in the army. By his election he became, in the faculty, the associate of Shippen, Wistar, and Rush, and worthily did he acquit himself of this honor as an able lecturer and successful experimenter. Dr. John Redman Coxe was his successor in 1809,—and afterwards carried his learning and research into the chair of materia medica, in 1818. Dr. John Syng Dorsey, early lost to the profession which he had already adorned, was made adjunct professor of surgery to his uncle, Dr. Physick, in 1807, and in 1816 received the appointment of professor of materia medica, as successor to Dr. Chapman, and in 1818 to that of anatomy, after the death of Dr. Wistar. He died in the latter part of the same year, leaving the chair to be filled by Dr. Physick, as it might have been filled by hundreds of inferior men. The change was ill-advised, and ought never to have been made. The great surgeon consented to place himself in a false position, under we know not what influences which were brought to bear on the occasion. Dr. Thomas C. James, whose wide range of reading and scholarship was veiled by his extreme modesty, was the first incumbent to the chair of midwifery, on its being recognized as a separate branch, in 1810.

Of Dr. Chapman, the successor of Dr. Barton, at first in the chair of materia medica, and then in that of the practice of medicine, we would gladly speak in terms becoming the merits of the man and the professor, were space left us for the purpose. A native of Virginia, he exhibited the urbane manners and pleasing colloquial powers joined to talents of a high order, which are so often met with in the inhabitants of that great State. He could see at once the strong points of a subject and present it with all the freshness of novelty. His practice and his teaching were for the most part eclectic; and theory was seldom hazarded except in explanation of co-ordinate or successive series of vital phenomena, in which he supposed sympathy to play an important part. He will be long and gratefully remembered as the founder of a summer school of medicine, known under the title of the "Philadelphia Medical Institute," and which had for its teachers and corporators Drs. Horner, Dewees, Jackson, Bell, J. K. Mitchell, Hodge, and Thomas Harris, in association with Dr. Chapman, and for assistants to some of these gentlemen, Drs. Goddard, Mütter, and Carson. The Institute was a nursery of professors; all of the parties just named, with the exception of Dr. Harris, having been elected to fill chairs in medical schools. Dr. Chapman was often spoken of as an indolent man, and to a casual acquaintance and superficial observer, who only saw him in his hours of ease—gay, smiling, and jocose—he might readily seem so. And yet, at the very time, when in the outer world in which he was daily moving, he might be thought by such persons to be doing nothing, he was editing and contributing articles to a medical journal, which he founded, and lecturing in the Medical Institute for five months every year, in addition to the discharge of duties of a similar nature, during nearly a like period, in the university: he was, also, attending, daily, a large circle of patients, and in a year of which we are now thinking, he brought out a new and enlarged edition of his work on Materia Medica and Therapeutics. For an indolent man, we never knew one who could interweave so much work with his indolence, if, in conformity with the received prejudices of the time, we still retain the term as at all applicable to his character.

We shall not trace the successive changes in the medical faculty of the university of later years, nor dwell on the lucid if not always elegant discourses of Dewees on midwifery, the painstaking and persevering industry of Horner in anatomy, and the genius of Hare, and the eloquence of James B. Rogers as teachers of chemistry. Nor is it our intention to give the history of the Jefferson Medical College nor of the Pennsylvania Medical College. The genius and surgical talent and the extensive reputation of the chief founder of both these institutions, Dr. George McClellan, and the rare ethnological knowledge of Dr. Samuel G. Morton, who for awhile taught anatomy in the latter, require a larger canvas and a freer pencil than we can command at the present time. In a history of the medical literature of Philadelphia, the labors of Dr. Eberle, another of the founders of the Jefferson Medical College, and the scientific attainments of Dr. Green, would find a prominent place. Dr. Granville Sharp Pattison, the attractive teacher of anatomy, will also be entitled to a full notice in any series of biographical sketches which shall be given of the professors in this school. For one session it was illumined by the flashing genius of Drake, who drew off with him to Cincinnati Drs. Eberle, Thos. D. Mitchell, and Gross. To the merits of its late eminent teacher and practitioner of surgery, Dr. Mütter, emphatic testimony was borne in the eloquent introductory lecture of a surviving colleague.* A similar and well-merited eulogy of the varied talents and contributions to medical science of Dr. John K. Mitchell, had been rendered in a previous year by his friend and successor in the chair of the practice of medicine.† In the Pennsylvania Medical College, tributes of respect and appreciative praise have been offered to the memory of Drs. Patterson, Bird, and Grant; the two former of whom taught, in succession, materia medica, the last, anatomy.‡

A century has elapsed since the Pennsylvania Hospital was founded, and in a few years more this period will be completed since the opening of the first medical school in Philadelphia. During this time the city has increased in population from 40,000 to 650,000 inhabitants. The hospital which received, in the first year after it was opened, ninety-nine patients,§ gave accommodation last year, 1858–9, to 1800, of whom about

* Dr. Pancoast. † Dr. Dickson.

‡ Dr. Samuel McClellan, once professor of midwifery in this institution, as he had been for awhile in the Jefferson Medical College, withdrew from the faculty some years before his death. The same remark applies to Walter Johnston, who was for a time professor of chemistry.

§ The number received in the provisional hospital, between February 11th, 1752, and May 4th, 1753, was sixty-four.

a third were surgical cases. The scanty provisions made, at the beginning, for the reception of the insane in one wing of the building have been expanded of late years to the erection, out of the city, of a large and separate structure, surrounded by extensive grounds, which affords means for the treatment of this class of patients beyond all former precedent, whether we look to other parts of the United States or even to Europe. The average number of patients during the year from May, 1858, to May, 1859, was 238. At the first commencement held by the medical faculty of the Philadelphia College, in 1768, ten of those who had attended lectures and complied with the required conditions previously laid down, received the degree of Bachelor of Medicine. At the commencement held in April, 1859, in the University of Pennsylvania, the degree of Doctor of Medicine was conferred on 144 candidates. In the Jefferson Medical College 256 received similar honors, as did also thirty-seven in the Pennsylvania College, and fourteen in the Philadelphia Medical College.* The entire number of graduates in 1859 goes beyond even what we may suppose to have been the most sanguine anticipations of Dr. Morgan in his introductory discourse, already quoted. In speaking of the future influence of the school, which he and his friend Dr. Shippen were about to found, he says: "By sending these abroad duly qualified, or by exciting an emulation among men of parts and literature, it may give birth to other useful institutions of a similar nature, or occasional rise, by its example, to numerous societies of different kinds, calculated to spread the light of knowledge through the whole American continent wherever inhabited." Words these of hope and of prophecy, which have been realized not only in the rise of rival institutions to the first school in Philadelphia itself, but of similar ones in nearly every section of our vast confederacy—extending from the Atlantic to the Pacific Oceans, and from the great lakes to the Gulf of Mexico. The number of students in attendance on the three medical schools in this city during the present winter, 1859–60, exceeds twelve hundred—a number beyond that collected in London, and, as we believe, we may say in Paris, not to speak of other European capitals.†

---

* This school has been closed; its faculty having taken chairs in the Pennsylvania Medical College, by an arrangement made with the gentlemen who had previously filled them, and under appointment by the Trustees at Gettysburg.

† The whole of the present paper was written for insertion in the January number of this Journal; but it was crowded out by other matter. The diminution in the number of students, chiefly at the expense of the Jefferson Medical College, by their sudden secession (abduction?) in the latter part of the month of December, 1859, without any complaint made against their professors, need only be adverted to on the present occasion.

Were we arguing a doubtful point, or were we merely intent on eulogizing the City of Philadelphia and her medical schools, we might now bring our narrative to a close, and leave our readers to a seemingly natural and satisfactory inference, viz., that one at least of the fountains of medical science is here perennial, and that the methods for distributing its waters to all sincere applicants athirst for knowledge have been, from year to year, for the last century, quite effective. We have dwelt with some emphasis on the singular merits of those who first opened out this spring, sacred to Esculapius and Hygeia. We leave to others to show that, with increased rate of distribution, there cannot be a falling off in the industry and ability of their successors, the present dispensers of the inspiring draughts. It does not comport with our intentions on the score of taste, to speak of the respective merits of the medical schools of this city, nor in any way of the personnel of their faculties, although we believe that few could do it with more impartiality. Our observations and sketches in this line have been of those who have passed away from the stage of life. If we should be tempted, at a future day, to discourse on the medical literature of Philadelphia, we would find it both our duty and pleasure to give names of professors who are authors, and to place them in a duly prominent point of view.

What are the means of instruction, material and intellectual, afforded to the medical students who, from all parts of the United States, have flocked to Philadelphia during the collegiate term of 1859–60? In the purely demonstrative branch of anatomy, that which lies at the foundation of correct medical knowledge, and the material for which can only be fully procured in a large city, there have always been the greatest facilities afforded both by the teaching of the professor in the amphitheatre and by dissections in the adjoining rooms under his direction, as well as in numerous private establishments under competent demonstrators. Chemistry, another branch largely demonstrative, is taught with all the apparatus demanded by the wants of modern science, and with limitations caused only by the short duration of the college session. Physiology, if not actually taught by experiment at the time, is made more and more to rest on the recorded and authentic results of experiments and experimental observation instituted elsewhere. The same may be said of pathology, which only fails to receive its fullest developments by the want of time allowed in the curricula of study. Continually, however, references are made to the statistical or numerical value of causes and to the results of actual dissection and to colored drawings of structural lesions—means these enlisted both by the professor of the institutes and the professor of the theory and practice of medicine. This branch, ceasing to be regarded as purely didactic, is now, in part at least, taught demonstratively, by the aids just mentioned and by imitations in *papier-maché* and composite material of a more enduring and indestructible nature. Surgery and midwifery are developed to the classes in descriptive prelections and in the exhibition of the use of the *armentaria*, as also through the aids furnished by the pictorial and plastic arts,—painting, charts, models. Materia medica is made familiar by the display of specimens of each article and often of the living plants, both indigenous and exotic, as introductory to an investigation of their therapeutic value. There may be differences in the number and richness of these various means of illustrating the lectures delivered in the schools, according to the taste and liberal expenditure of the professors; but no essential advance in this respect can be made in one of them which is not imitated by the others.

In one particular there is a general accordance of sentiment and action among the faculties of our three medical schools, viz., in the prominence given to clinical instruction. Philadelphia, although one of the healthiest cities in the world, must necessarily, owing to her large population, furnish ample materials for this purpose—derived from the sailors who frequent her port and the indigent sick, as well native as foreign. Hospitals, infirmaries, and dispensaries give temporary refuge and present relief to the ailments of those parties, and supply them at the moment with the needful medicines; and while thus discharging their original functions under the prompting of charity, they furnish, at the same time, the best opportunities for obtaining familiarity with diseases in all their original varieties and subsequent proteiform changes. Foremost among these institutions stands the Pennsylvania Hospital, in which the intelligent student, after seeing and examining the sick occupants of its beds, will listen to the clinical remarks of the attending physician and surgeon, and thus be made familiar with the causes and incidents of disease and the doubts and difficulties of bedside practice in his own future career. To aid him in a further solution of the problem he can, on retiring, take with him a volume from the rich treasures of medical lore in the library of the hospital, and thus compare the experience of an earlier teacher with the observations of the present time. Similar advantages, on a still larger scale, are offered in the extensive wards of the Blockley Hospital, connected with the Alms House, on the western side of the Schuylkill River. Of late years, opportunities for acquiring clinical knowledge have been afforded in the provisional buildings of two new hospitals, the Protestant Episcopal and St. Joseph's. An ample structure for the former will, we understand, be ere long erected, and there are not wanting zeal and liberality to place the latter on an equally stable foundation. Wills' Hospital for Diseases of the Eyes and Limbs bespeaks its objects by its title. So also with the Children's Hospital. The Western Clinical Infirmary, chartered a few years ago, contains fifty beds: it has received the donation of a lot of ground, and measures are in progress for erecting on it a hospital, to be called after the great philanthropist Howard. "The Home for Invalids with Diseases of the Chest," furnishes the means of observing the symptoms and progressive changes of the chronic affections of these organs, especially phthisis. The Philadephia Dispensary, the oldest institution of its kind in the city, presents a good school for the observation of disease in all its chronic and subacute forms, and also for the study of minor surgery. The Northern Dispensary affords similar benefits, both in a curative and clinical point of view.

Each of the medical schools has its dispensary, and limited hospital accommodations. In the first a large number of patients, requiring medical and surgical assistance, is examined and prescribed for, and, when occasion demands, operated on before the class, every Wednesday and Saturday. Each of the two professors on duty at the time takes those cases which come under the heads respectively of medicine and surgery. Patients who are made the subjects of capital operations in the amphitheatre are placed in the beds prepared for the purpose, in the college building. One advantage of these exhibitions is to make the student familiar with the methods of examination and inquiry into the symptoms and causes of the disease, when it is first seen, and of the general outlines of treatment; but the details of which, as demanded in the progress of the case, he is of course unable to follow.

In addition to the means of instruction afforded within the walls of the colleges, availing themselves of which on the part of the students registered in each is an indispensable condition for their receiving the honors of the doctorate, there are others both numerous and valuable offered by private lecturers and teachers on nearly every department of medical science and divisions of medical practice. Of these we may mention practical or demonstrative courses on anatomy, obstetrics, pathological anatomy, microscopic anatomy, operative surgery, pharmacy and materia medica, diseases of the chest, diseases of the lungs and heart, medicine and surgery of the eye and ear: also, lectures on the practice of medicine, instruction in analytical chemistry in the laboratory, and instruction in dental surgery. Examinations in the several subjects taught in the three schools are conducted by quite a number of gentlemen; two, three, or more of whom are associated with each other for the purpose, during the winter.

In conclusion, we would remark, that, although the primary motive for medical students coming to Philadelphia is to attend the lectures delivered in the school of their choice, and the subsequent paramount consideration with them is to obtain a degree, they cannot but be conscious of the value of the collateral and auxiliary means procurable by them for more minute investigations with the aid of a private lecturer, who has made the subject his specialty. For example, histology, physiological and pathological, analytical chemistry in its relations to toxicology and pathology, pharmacy, diagnosis of diseases of the chest by physical exploration, minor and operative surgery, practical obstetricy and study of the organic diseases of females, are learned with most advantage in private courses, in which the students can inspect closely, touch and manipulate for themselves, under the direction of their instructor for the time being. It is true that these numerous appliances for facilitating the acquisition of both theoretical and practical medicine cannot be turned to much immediate account by the great body of students who constitute the classes during their attendance at the different schools, owing to the want of time, pressed as they are to follow, from hour to hour, the several courses of the professors, and feeling themselves obliged to occupy the little time left in a revision of their notes taken in the lecture-room, or, preferably, in a reference to their text-books or other works germain to their immediate subject. But granting all due weight to this fact, it must be a gratifying reflection to the studious crowd, at the end of the winter or regular session, that to those of their number who are intent on a still more intimate acquaintance with one or more of the branches just named, the wished-for opportunities and facilities are at hand. They who are disposed to prolong their stay in the city, or to remain during the entire interval between the regular collegiate courses, will be able to avail themselves of continued clinical instruction in the Pennsylvania and Blockley Hospitals, and, being less pressed for time, they can look, also, into the wards of St. Joseph's and the Protestant Episcopal. A portion of this period will be given to regular attendance on the lectures and demonstrations which can be carried on without interruption during the season. The bill of fare for intellectual nourishment and gratification is varied, and is, at the same time, adapted to the different wants and tastes of the students, according to their previous progress or ultimate views of the branch to which they propose most to addict themselves. They will, if they remain in the city during the entire recess, feel, even still more than when attending the winter lectures in the schools, that they breathe a medical atmosphere, and will learn, in some measure, the reasons, in addition to the abilities of their public teachers, for the prominence which Philadelphia maintains as the great centre of medical education in the United States.

We close this paper with an extract from one of the articles in the *North American and United States Gazette* on our present subject, under date of September 15th, 1856, while cheerfully adopting as our own the sentiments contained in it. The writer, after quoting from Dr. Morgan's discourse respecting the advantages which would accrue to Philadelphia in a pecuniary point of view, continues in the following strain:—

"Other results of a still more gratifying kind, which are not reducible to a pecuniary valuation, ensue from the social relations established between the students of medicine and our citizens, by intimacies which often settle down into enduring friendships, and sometimes lead to a connection of a still more endearing character. For the most part, however, such is the continued strain on the mind of the student, by his attendance on the different lectures and on hospital and other clinical practice, and reading of text-books and works of reference, that he can scarcely allow himself any time for paying or receiving visits or enjoying the pleasures of society.

"Here we may be allowed to assert advisedly that, as a class, the medical students who annually resort to our city, including many who remain here during the whole year, will compare advantageously with the same number of young men selected from the wealthiest and best educated families in any community; and, in persevering and methodical study, to the sacrifice of their comfort, and too often of their health, they go far beyond them. It used to be the fashion to feel or affect a certain amount of dread at the frolics and pranks of the 'Virginia doctors,' a title given to medical students, whether they hailed from Maine or from Louisiana. But if a few wild, and, perhaps, dissipated characters were to give a name to a body of more than a thousand students, young and inexperienced, and for the first time their own masters and their own treasurers, we might, bringing the same rule of proportion to bear, have serious apprehensions of the probity of the trading classes and the piety of our regular church-goers. A slight infraction of the law, or even a serious *melée*, in which a student happens to be the offender, obtains a publicity, in a police report, which a young scapegrace of a citizen, similarly circumstanced, would avoid by the ready bail and propitiatory intervention of a father or other relative or friend. We should be doing injustice to the professors of our different schools, were we to omit to say that they are always ready, so soon as they hear of one of their students being involved in any difficulty, to interpose with their good offices, and to save him from pains and penalties as far as the most liberal construction of the law will allow. It would hardly be worth while to advert to this subject at all, but for the misconceptions sometimes entertained at a distance, in consequence of misrepresentations and exaggerations which we are afraid cannot, in every case, be attributed to ignorance. As regards harmony among the members of the medical classes, we cannot but deem it a peculiarly fortunate circumstance and an evidence both of the sober judgment and good taste of the professors and of the discretion of the students themselves, that from the first medical commencement, in 1768, down to the present day, embracing a period of eighty-seven years, the lecture-rooms have never been the arena of political, sectarian, or sectional discussions or declamations, either on the part of teachers or pupils. Philadelphia, during six months of the year, is the seat of a medical congress, the members of which, students, are delegates for the nonce from nearly every part of the United States. They come on for a specific purpose, and they have neither the time nor the inclination to attend to, or even to think of foreign, and barely of collateral issues. They distribute themselves in the different schools, under the professors whom they prefer, sure that these latter will never obtrude on them their political preferences, their religious creeds, or their crotchets, if they have any, in philosophy or ethics. If, outside of the college walls, the students witness, by chance, a party procession with its banners and divers mottoes, they may regard it as they would any other show, which, once passed, leaves them undisturbed to pursue the even tenor of their way. Sometimes it may happen that, in their quizzing clubs, at their own rooms, after they have examined each other on the several subjects taught in the college a day or two or three days previously, they will, apropos of the mention of a name high in politics, or at the bar, or in the church, and, especially if he be a popular speaker, discuss his merits. On these occasions there is a very natural leaning on the part of each member in favor of the great man of his State or district, and discussions, in a friendly spirit, sometimes originate in consequence."*

Among the medical institutions of Philadelphia, which do not belong to the educational class, and which are composed of graduated and practicing physicians, are the College of Physicians, and the Philadelphia County Medical Society. The first was incorporated in 1789, with a design, as stated in its charter, to advance the science of medicine by steps therein specified, which include a cultivation of order and uniformity in the practice of physic. The Philadelphia County Medical Society was founded in 1849: it is in more immediate connection with the Pennsylvania State Medical Society, by its being empowered to send delegates annually to the meetings of this latter. The old Philadelphia Medical Society, which was instituted in 1789 and incorporated in 1792, was chiefly a debating society, in which papers were read and discussed by the senior members, who were graduates, in the presence and for the benefit of junior members, who as students of medicine and attending lectures had been introduced, after examinations, by a committee appointed for the purpose. This Society, which had been in a great measure dormant for several years, has of late been revived, and at the same time undergone some modifications of its constitution and by-laws.

Resting in a great measure on a scientific basis, and with a purpose indicated by its title, is the Pathological Society of Philadelphia, which, although of recent origin, has already issued many papers and observa-

---

* We are sure that Mr. McMichael, the proprietor and able chief of the editorial corps of the *North American and United States Gazette*, will not object to our large borrowing, in other parts of this article, from the columns of his influential and conservative paper, without specific acknowledgment. We would add the fact, as coming within our own knowledge, that the whole subject was suggested by him, in his never-failing desire to advance the interests and the reputation of Philadelphia, by exhibiting both her material and intellectual capabilities and resources.

tions of marked worth. It ought to have been a branch, in the form of a Standing Committee, of the College of Physicians—as the Biological Society is of the Academy of Natural Sciences. This last-named body and its new progeny, while cultivating the large domain of natural history and comparative anatomy and physiology, cannot fail to shed light on some of the yet intricate points of human physiology. The Academy has a rich museum and a large and valuable library.

As it did not form any part of our plan to give the history, even in its most abbreviated form, of the medical vagaries of opinion and teaching in Philadelphia, as exhibited in the promulgation of particular creeds, remarkable either for the extravagance of their appeals to credulity and the absence of all the ordinary rules of evidence, or for a limited, one-sided, and exclusive restriction to a part only of the recorded experience based on demonstration in all periods of civilization, and in all climates and circumstances, we have not thought it worth while to make any mention, even in the way of stricture, of the associations or pseudo-schools which inculcate such medical heresies and foster a restless and impatient spirit and ignorant self-sufficiency, adverse to sober reasoning on the largest number of facts, as well as to sound learning and prolonged study and meditation. These last-mentioned requirements for solving difficult problems in science are considered of small moment by the volunteer medicasters, who tamper with the human frame with a freedom and recklessness that would not be thought of, or, if tried, tolerated in the case of the simplest form of mechanism of inorganic matter. The easy, good nature of well-educated men, and some of them members of other professions, is much more conspicuous than their conscientiousness and judgment, when they allow their names to be placed among the trustees or directors of hybrid medical schools, the cardinal doctrines of which rest on fallacies of observation as well as of induction, perilous to human health and human life. We have, already, in a former number of this Journal, expressed our opinions on the subject of women practicing physic, and we see no reason to change or qualify them on the present occasion.

